# Apolipoprotein E regulates lipid metabolism and α-synuclein pathology in human iPSC-derived cerebral organoids

**DOI:** 10.1007/s00401-021-02361-9

**Published:** 2021-08-28

**Authors:** Jing Zhao, Wenyan Lu, Yingxue Ren, Yuan Fu, Yuka A. Martens, Francis Shue, Mary D. Davis, Xue Wang, Kai Chen, Fuyao Li, Chia-Chen Liu, Neill R. Graff-Radford, Zbigniew K. Wszolek, Steven G. Younkin, David A. Brafman, Nilüfer Ertekin-Taner, Yan W. Asmann, Dennis W. Dickson, Ziying Xu, Meixia Pan, Xianlin Han, Takahisa Kanekiyo, Guojun Bu

**Affiliations:** 1 Department of Neuroscience, Mayo Clinic, Jacksonville, FL 32224 USA; 2 Center for Regenerative Medicine, Neuroregeneration Laboratory, Mayo Clinic, Jacksonville, FL 32224 USA; 3 Department of Health Sciences Research, Mayo Clinic, Jacksonville, FL 32224 USA; 4 Department of Neurology, Mayo Clinic, Jacksonville, FL 32224 USA; 5 School of Biological & Health Systems Engineering, Arizona State University, Tempe, AZ 85287 USA; 6 Barshop Institute for Longevity and Aging Studies, University of Texas Health Science Center At San Antonio, San Antonio, TX 78229 USA; 7 Department of Medicine, University of Texas Health Science Center At San Antonio, San Antonio, TX 78229 USA

## Abstract

*APOE4* is a strong genetic risk factor for Alzheimer’s disease and Dementia with Lewy bodies; however, how its expression impacts pathogenic pathways in a human-relevant system is not clear. Here using human iPSC-derived cerebral organoid models, we find that *APOE* deletion increases α-synuclein (αSyn) accumulation accompanied with synaptic loss, reduction of GBA levels, lipid droplet accumulation and dysregulation of intracellular organelles. These phenotypes are partially rescued by exogenous apoE2 and apoE3, but not apoE4. Lipidomics analysis detects the increased fatty acid utilization and cholesterol ester accumulation in apoE-deficient cerebral organoids. Furthermore, *APOE4* cerebral organoids have increased αSyn accumulation compared to those with *APOE3*. Carrying *APOE4* also increases apoE association with Lewy bodies in postmortem brains from patients with Lewy body disease. Our findings reveal the predominant role of apoE in lipid metabolism and αSyn pathology in iPSC-derived cerebral organoids, providing mechanistic insights into how *APOE4* drives the risk for synucleinopathies.

**Supplementary Information:**

The online version contains supplementary material available at 10.1007/s00401-021-02361-9.

## Introduction

α-synuclein (αSyn) encoded by *SNCA* is a presynaptic membrane-bound protein abundantly expressed in the brain [3, 60]. The aggregated forms of αSyn often accumulate in neurons as Lewy bodies which are the primary pathological features of synucleinopathies [49, 58] in several neurodegenerative diseases including Parkinson’s disease (PD), Parkinson’s disease dementia (PDD), and Dementia with Lewy bodies (DLB) [3, 28, 43, 49]. Lewy body disease is one of the most common causes of dementia after Alzheimer’s disease (AD) and vascular dementia [10, 17]. Indeed, DLB alone is predicted to account for approximately 5% of dementia cases, although AD pathology is frequently detected in DLB brains [1, 3]. Notably, accumulating genetic evidences indicate the substantial contributions of genetic risk factors to the disease. A recent genome-wide association study has confirmed the associations of *APOE*, *GBA* and *SNCA* gene variants with DLB [19]. However, there is still a gap in our knowledge regarding how these genes contribute to the pathogenesis of DLB.

In humans, the *APOE* gene encoding apolipoprotein E (apoE) exists as three polymorphic alleles (*APOE2*, *APOE3*, and *APOE4*), where *APOE4* is the strongest genetic risk factor for AD [31, 36]. Furthermore, *APOE* genotype has been shown to be associated with the progression or poor clinical outcomes of other neurological or neurodegenerative diseases [62, 68]. Importantly, *APOE4* is not only associated with the risk for DLB, PDD and cognitive impairment in PD [29, 35, 61], but also the increased severity of synucleinopathies independent of amyloid pathology in AD [11]. ApoE mediates multiple pathways in the maintenance of brain homeostasis, including lipid transport, synaptic integrity and plasticity, and glucose metabolism [37, 67, 68, 73]. Since cellular membranes are mainly composed of cholesterol and phospholipids, lipid metabolism via apoE-containing lipoproteins is likely involved in synaptogenesis [39] as well as membrane trafficking [27] in neurons. Thus, greater understanding of the physiological role of apoE in the central nervous system is critical for uncovering the underlying pathogenic mechanisms in which *APOE4* aggravates synucleinopathies.

In this study, we investigate how apoE deficiency impacts αSyn pathology and lipid metabolism using the human induced pluripotent stem cells (iPSC)-derived cerebral organoid models from cognitively unimpaired individuals. The three-dimensional (3-D) cerebral organoids generated from iPSCs are useful tools to model neurodegenerative diseases, because they display intrinsic spatial patterning and cell diversity with sequential neuronal layer formation, accompanied with astrocyte maturation [32, 51, 54]. We show that *APOE* deletion facilitates the accumulation of insoluble αSyn in iPSC-derived cerebral organoids. Increased lipid droplet accumulation and synaptic loss are also detected in apoE-deficient cerebral organoids compared to those from isogenic controls. Remarkably, the phenotype can be partially alleviated by exogenous astrocytic apoE2 and apoE3, but not apoE4. Consistently, carrying homozygous *APOE4* increases αSyn accumulation and lipid droplet formation in the cerebral organoids compared with those with homozygous *APOE3*, with positive correlations detected between insoluble apoE and αSyn levels. Postmortem brains from Lewy body disease carrying *APOE4* also show increased apoE accumulation in Lewy bodies. Together, our findings using human cerebral organoid models demonstrate that apoE isoforms contribute to αSyn pathology.

## Online methods

### Generation of iPSCs from human skin fibroblasts

Human skin biopsies from normal individuals with *APOE* ε3/ε3 or ε4/ε4 genotype were obtained with patient consent from Mayo Clinic under IRB protocols approved by the Mayo Clinic Institutional Review Board. *APOE* genotype was confirmed by Sanger sequencing using DNA samples from fibroblasts. Cells were cultured in fibroblast medium containing 10% fetal bovine serum (FBS) (Gemini Bio-Products). Three episomal vectors were electroporated into the fibroblasts using the NHDF nucleofector kit (Lonza) [48, 66]. 6 × 10^5^ fibroblasts were transfected with 3 μg of expression plasmid mixtures with 100 μl transfection reagents. Fibroblasts were then plated onto a 100 mm Matrigel (Corning) coated dish. Fibroblast medium was replaced with TeSR-E7 complete medium (Stemcell Technologies) after 5 days. Daily medium change was applied for 3–4 weeks. iPSC colonies were isolated and expanded for further characterization. The iPSC colonies were passaged using Dispase (Stemcell Technologies) and subjected to rock inhibitor Y27632 (Sigma-Aldrich) treatment for the first 24 h.

### Generation of *APOE*^−/−^ iPSCs

Two different sets of human parental iPSC line and its isogenic *APOE*^*−/−*^ iPSC line were used in the study. The first set of iPSC lines were obtained from Xcell Science (XCL-1 and XCL-*APOE* knockout), isogenic *APOE*^*−/−*^ iPSC line was generated via Zinc Finger Nucleases (ZFN) gene editing method. Further validation information about this line can be found in the Xcell Science website (http://www.xcellscience.com/products/ipsc). For the second set of iPSC lines (MC0192 and MC0192-*APOE* knockout), isogenic *APOE*^*−/−*^ iPSC line was obtained via CRISPR/Cas9 gene editing method by ALSTEM. Two gRNA/Cas9 constructs for human *APOE* were designed to target exon2 of *APOE* gene [*APOE* gRNA5: GGCCAAGGTGGAGCAAGCGG (TGG); *APOE* gRNA8: ACAGTGTCTGCACC CAGCGC (AGG)]. Mc0192 iPSCs were cultured in complete mTeSR1 media plus Pen/Strep antibiotics at 37 °C with 5% CO2. About 3 × 10^5^ cells were transfected with 1.5 μg of each gRNA/Cas9 plasmids by Invitrogen Neon transfection system. After transfection, cell lysis was used to examine the knockout efficiency by PCR (primers: *APOE*-F AGGTACTAGATGCCTGGACGG; *APOE*-R GTATAGCCGCCCACCAGGAG). Single cells were plated in multiple 96-well plates and cultured for 14 days before expanding to 12 well plates. Genomic DNA was subsequently extracted from single cell clones and used for PCR analysis for identifying knockout clones. Knockouts were further confirmed by DNA sequencing.

### Cerebral organoid culture

STEMdiff™ Cerebral Organoid Kit (Stemcell Technologies) was used to generate cerebral organoids following manufacturer’s instructions. On Day 0, iPSC colonies were dissociated into single cell suspension with Accutase, where 15,000 cells were seeded into a U-bottom ultra-low-attachment 96-well plate in EB formation media (medium A) supplemented with 10 μM Y-27632. On Day 2 and Day 4, additional 100 μl of medium A were added per well. On Day 5, EBs were moved to 48-well low attachment plates in neural induction medium (medium B) and left for an additional 3–5 days. EBs were further embedded into 20 μl of matrigel and cultured in neural expansion medium (medium C + D) for 3 days in 6-well low attachment plates for organoid formation. Finally, organoids were transferred to 10 cm dishes and moved to an orbital shaker for further culture in neural culture medium (medium E). After 4 weeks, medium E was replaced with neuronal maturation medium consisting of: DMEM/F12 + Neurobasal Medium (1:1) supplemented with N2, B27, BDNF (20 ng/ml), GDNF (20 ng/ml), ascorbic acid (200 μM) and dbcAMP (100 nM) (Sigma Aldrich) [47]. Cerebral organoids were harvested at pre-defined time points for immunostaining and biochemical analysis.

### Tissue processing

Cerebral organoids were harvested at pre-defined time points and lysed with RIPA lysis and extraction buffer supplemented with protease and phosphatase inhibitor cocktails for cell lysis (Roche). Samples were kept on ice for 60 min after sonication, and then centrifuged in an ultracentrifuge (Beckman-Coulter) at 100,000*g*, for 1 h at 4 °C. Supernatants were collected and labeled as the soluble fraction. The pellets were resuspended and sonicated in 2% SDS buffer (Sigma-Aldrich) and then centrifuged at 50,000*g* for 20 min at 4 °C. The supernatants were collected as SDS fractions. Total protein concentration in the soluble fraction was determined using a Pierce BCA Protein Assay Kit.

### Immunostaining and BODIPY staining of cerebral organoids

At Day 30 and Day 90, cerebral organoids were harvested and fixed in 4% paraformaldehyde for 30 min then washed with PBS three times. After fixation, organoids were dehydrated with 30% sucrose in PBS at 4 °C. Optical cutting temperature (OCT) compound (VWR) was used to embed cerebral organoids, which were subsequently frozen on dry ice. Tissue was sectioned using a cryostat at 30 μm. Sections were collected on ultra-frosted glass microscope slides and stored at − 20 °C. For immunostaining, sections were permeabilized in 0.25% Triton X-100 and blocked with blocking buffer containing 4% normal donkey serum, 2% BSA and 1 M glycine in PBS. Sections were then incubated with primary antibodies in blocking buffer overnight at 4 °C. Primary antibodies and their dilutions used in this study are as follows: Sox2 (Abcam, ab97959, 1:500), Tuj1 (Abcam, ab78078, 1:1000), Tuj1 (Sigma, T2200, 1:1000), GFAP (Millipore, MAB360, 1:300), αSyn 4B12 (Biolegend, 807801, 1:300), and MJFR14 (Abcam, ab209538, 1:300) On the following day, sections were washed three times with PBS, and then incubated with secondary antibodies for 2 h at room temperature. Finally, sections were washed three times with PBS before mounting with the glass coverslip. Secondary antibodies and their dilutions used in this study are as follows: Alexa Fluor donkey anti-rabbit 488, 594 (Invitrogen, A32790, A32754, 1:500), and Alexa Fluor donkey anti-mouse 488, 594 (Invitrogen A32766, A32744, 1:500). For BODIPY staining, sections were washed three times in PBS and incubated in PBS with BODIPYTM 493/503 (1:1000 from 1 mg/ml stock solution in DMSO; ThermoFisher) to stain lipid droplets and DAPI (1:4000, ThermoFisher) for nuclear counterstaining for 5 min at RT. Sections were washed three times in PBS and mounted with Vectashield (H-1000, Vector Laboratories). Fluorescent signals were detected by Keyence fluorescence microscopy (model BZ-X, Keyence) and quantified using ImageJ software.

### Western blotting

RIPA soluble and SDS soluble fractions collected from cerebral organoids were run on a 4–20% sodium dodecyl sulfate–polyacrylamide gel (Bio-Rad), and transferred to PVDF Immobilon FL membranes (Millipore). Membranes were blocked in 5% non-fat milk in PBS and subsequently incubated overnight with primary antibodies in 5% non-fat milk/PBS containing 0.1% Tween-20. Primary antibodies and their dilutions used in this study are as follows: ApoE (Meridian Life Science, K74180B, 1:1000), cleaved CASP3 (Cell Signaling Technology, 9661, 1:1000), CASP3 (Cell Signaling Technology, 9662, 1:1000), PSD95 (Abcam, ab2723, 1: 1000), SYP (Abcam, ab8049, 1:1000), Tuj1 (Sigma, T2200, 1:4000), GFAP (Millipore, MAB360, 1:1000), EEA1 (Cell Signaling Technology, 2411, 1:1000), LAMP1 (Cell Signaling Technology, 9091, 1:1000), Vps39 (Abcam, ab224671, 1:1000), Plin2 (R&D, MAB7634, 1:1000), GCase (Abcam, ab175869, 1:1000), LIMP1 (Abcam, ab59479, 1:1000), αSyn 4B12 (Biolegend, 807801, 1:1000), αSyn (Cell Signaling Technology, 2642 s, 1:1000), p-αSyn (Cell signaling, 23706S, 1:1000), p-αSyn (Invitrogen, MA5-34,671, 1:1000), and β-actin (Sigma, A2228, 1:4000). On the following day, membranes were probed with LI-COR IRDye secondary antibodies or horseradish peroxidase-conjugated secondary antibody, detected with SuperSignal West Femto Chemiluminescent Substrate (Pierce). The specificities of αSyn 4B12 and p-αSyn antibodies to the SDS fraction samples were confirmed using their specific blocking peptides (recombinant αSyn, Anaspec, AS-55555; p-αSyn-S129 peptide, Abcam, ab188826) (Supplementary Fig. 1, online resource).

### RT-qPCR

Trizol/chloroform method was used to extract RNA from organoids, followed by DNase and cleanup using the RNase-Free DNase Set and the RNeasy Mini Kit (QIAGEN). The quantity and quality of all RNA samples for RNA-seq was determined by the Agilent 2100 Bioanalyzer using the Agilent RNA 6000 Nano Chip (Agilent Technologies, CA). The cDNA was prepared with the iScript cDNA synthesis kit (Bio-Rad). Real-time qPCR was conducted with Universal SYBR Green Supermix (Bio-Rad) using an iCycler thermocycler (Bio-Rad). Relative gene expression was normalized to *ACTB* gene coding β-actin and assessed using the 2 − ∆∆CT method. Primers used to amplify target genes by RT-qPCR are as follows: *ACTB* F (5’-CTGGCACCACACCTTCTACAATG-3’) and R (5’-AATGTCACGCACGATTTCCCGC-3’), *APOE* F (5’-CGTTGCTGGTCACATTCCT-3’) and R (5’-CTCAGTTCCTGGGTGACCTG-3’), *GBA* (PPH15870B, QIAGEN).

### Glucocerebrosidase activity assay

The GCase enzyme activity was measured using the Glucosylceramidase Activity Assay Kit (Fluorometric, ab273339) according to the manufacturer’s instructions. Cerebral organoids were homogenized with the homogenization buffer and the cell pellet was disrupted on ice with a probe sonicator. The cell lysate was centrifuged at 12,000*g* for 10 min at 4 °C with protein concentration quantified using a BCA assay. In a 96-well plate, 2–20 μl cell lysate was added and adjusted to 40 μl with Glucosylceramidase assay buffer. Then 20 μl substrate was added and incubated at 37 °C for 30 min in dark. The reaction was stopped with 100 μl stop buffer and the fluorescence was read at 360 nm excitation 445 nm emission.

### Aβ ELISA

The Aβ40 and Aβ42 in both RIPA and SDS fractions were measured using the Human β-Amyloid (1–40) ELISA Kit (ThermoFisher, KHB3481) and Human β-Amyloid (1–42) ELISA Kit (ThermoFisher, KHB3441) according to the manufacturer’s instructions. 50 µL of standards/pre-dilute samples and 50 µL of Detection Antibody solution were added to the appropriate wells. The plate was covered and incubated at 4 °C overnight. The plates were washed four times with wash buffer and incubated with 100 µL Anti-Rabbit IgG HRP for 90 min at room temperature. After washing four times with wash buffer, 100 µL Stabilized Chromogen was added and incubated for 30 min at room temperature in dark. The reaction was stopped and read at 450 nm with a microplate reader (Biotek). Results were normalized to total protein concentration of the corresponding cell lysate.

### RNA-seq, quality control and normalization

Six mRNA samples were sequenced at Mayo Clinic sequencing core using Illumina HiSeq 4000. Reads were mapped to the human reference genome hg38. Mayo Clinic RNA-Seq analytic pipeline (MAP-RSeq Version 3.0) was used to generate raw gene read counts and conduct sequencing quality control [30]. Raw gene counts were corrected for gene length differences, GC bias, global technical variations via Conditional Quantile Normalization (CQN) to obtain similar quantile-by-quantile distributions of gene expression levels across samples [22]. Based on the bi-modal distribution of the CQN normalized and log2-transformed reads per kb per million (RPKM) gene expression values, genes with an average of log2 RPKM >  = 3 in at least one group were considered expressed. With this threshold, 17,810 genes were identified for downstream analysis.

### Differential gene expression

Principal component analysis (PCA) and differential expression analysis were performed using the Partek Genomics Suite (Partek Inc., St. Louis, MO). ANOVA was used to compare gene expression between parental and *APOE*^*−/−*^ isogenic cerebral organoids while correcting for the effects of RNA integrity number (RIN). Volcano plots of differentially expressed genes were generated using R version 3.4.1.

### DEGs cell-type distribution analysis of RNA-seq data

Cell proportion was estimated using the CIBERSORT program [45] and marker genes described in BRETIGEA [42]. Within the top 50 marker genes of neuron, astrocyte and oligodendrocyte cells obtained from BRETIGEA [42], 30 neuronal markers, 27 astrocytic markers and 19 oligodendrocyte markers were found in both reference dataset and our iPSC dataset. Expression levels of these markers in sorted neurons, astrocytes and oligodendrocytes were further obtained from the reference dataset of Zhang et al. [70]. To estimate cellular composition in the iPSC dataset, CIBERSORT analytical tool [45] was applied based on the selected maker gene expression in reference dataset and in the iPSC dataset for neurons, and astrocytes. CellCODE R package [7] was applied to assess the interaction between group variable and surrogate proportion variables of each cell type, which further helped us to estimate which cell type genes might be regulated. Specifically, getAllSPV function in CellCODE refined the previously stated top 50 marker genes from BRETIGEA and obtained surrogate variables of neuron and astrocyte populations using the remaining markers through singular value decomposition. CellPopT function was then used to calculate the t statistics of the interaction term between group and surrogate variables. Cell type with the largest t-statistics was defined as the designated cell type.

### WGCNA

Weighted Gene Co-expression Network Analysis (WGCNA) [33] was applied to identify groups of genes that are correlated with *APOE*, using the log2-transformed, CQN-normalized gene expression values after correction of RIN. The soft power of 16, hybrid dynamic tree cutting, a minimum module size of 50 genes, and a minimum height for merging modules at 0.3 was used to build a signed hybrid co-expression network. Each gene module was summarized by the first principal component of the scaled module expression profiles (module eigengene). A unique color identifier was assigned to each module, and genes that did not fulfill these criteria were assigned to the gray module. Parental cerebral organoids were defined as 0 and *APOE-/-* cerebral organoids were defined as 1 to assess the correlation of modules to *APOE*. R package anRichment (https://horvath.genetics.ucla.edu/html/ CoexpressionNetwork/GeneAnnotation/) was used to annotate different modules. VisANT was applied to visualize the connection among the top hub genes in the module [26].

### Lipidomics

Lipid species were analyzed using multidimensional mass spectrometry-based shotgun lipidomic analysis [20]. In brief, the cell pellets were homogenized with 0.1 × PBS at 6500 rpm, 0 °C in 2 ml Precellys Lysing Kit (Bertin, France) using Cryolys Evolution homogenizer (Precellys^®^ Evolution, USA). The cell pellet homogenate containing 0.8 mg of protein which was determined with a Pierce™ BCA protein assay kit (Cat #23225, Thermo Scientific) or 2 mL of cell culture medium was accurately transferred to a disposable glass culture test tube, respectively. A premixture of lipid internal standards (IS) was added prior to conducting lipid extraction for quantification of the targeted lipid species. Lipid extraction was performed using a modified Bligh and Dyer procedure [63], and each lipid extract was reconstituted in chloroform: methanol (1:1, v:v) at a volume of 400 µL/mg protein or 80 µL/mL culture medium.

For shotgun lipidomics, lipid extract was further diluted to a final concentration of ~ 500 fmol total lipids per µL. Mass spectrometric analysis was performed on a triple quadrupole mass spectrometer (TSQ Altis, Thermo Fisher Scientific, San Jose, CA) and a Q Exactive mass spectrometer (Thermo Scientific, San Jose, CA), both of which were equipped with an automated nanospray device (TriVersa NanoMate, Advion Bioscience Ltd., Ithaca, NY) as described [21]. Identification and quantification of lipid species were performed using an automated software program [64, 69]. Data processing (e.g., ion peak selection, baseline correction, data transfer, peak intensity comparison and quantitation) was performed as described [69]. The result was normalized to the protein content (nmol lipid/mg protein) or the culture medium volume (nmol lipid/mL medium), respectively. Differential expression analysis was performed using the Partek Genomics Suite (Partek Inc., St. Louis, MO). ANOVA were used to compare lipid profiles between parental and isogenic *APOE*^*−/−*^ samples using log transformed and normalized values at day 30 and day 90, respectively. Hierachical clustering analysis was used to visualize the top 50 differentially expressed lipids at day 30 and day 90.

### Immortalized astrocyte culture and preparation of conditioned medium

Immortalized astrocytes (*APOE2*, *APOE3*, and *APOE4*) were a kind gift from Dr. David M. Holtzman (Washington University). These cell lines were generated from primary astrocytes derived from human *APOE*-TR mice, in which human *APOE* gene is knocked in the mouse *Apoe* locus [44]. Immortalized *Apoe-*knockout astrocytes were generated in house from primary astrocytes derived from *Apoe-*knockout mice following published protocol [44]. Immortalized astrocytes with different *APOE* genotypes (*APOE2*, *APOE3*, *APOE4* and *Apoe*-knockout) were maintained in 175 cm^2^ flasks in Dulbecco’s modified Eagle’s medium/Ham’s F-12 containing 20% FBS (Hyclone), 100 units/ml penicillin, 100 μg/ml streptomycin, and 250 ng/ml fungizone. When astrocyte culture reached confluency, cells were rinsed two times with phosphate-buffered saline (PBS, pH 7.4) followed by adding serum-free, Dulbecco’s modified Eagle’s medium/Ham’s F-12 containing 1% N-2 supplement (Invitrogen), 100 units/ml penicillin, 100 μg/ml streptomycin, and 250 ng/ml fungizone. Conditioned medium was harvested after 48 h and filtered through 0.45 μm membrane, ApoE concentration in *APOE2*, *APOE3* and *APOE4* conditioned medium was adjusted to 1 µg/ml using *Apoe*-knockout conditioned medium. 50 ml conditioned medium was then concentrated 100 times via Amicon Ultra-15 Centrifugal Filter Units (10 KD cutoff, Millipore), aliquoted, and stored in − 80 °C until further use.

### Treatment of cerebral organoids with conditioned media

Cerebral organoids at Day 90 were subjected to a full medium change by adding 15 ml neuron culture medium in each 10 cm culture dish. Immortalized astrocytes conditioned medium was added at a ratio of 1:100 (adding 150 µl concentrated medium into 15 ml neuron medium). Cerebral organoids were harvested after 5 days for further analysis.

### Cholesterol assay in cerebral organoids

The amount of cholesterol in the RIPA fraction of cerebral organoids was determined with the Amplex Red cholesterol assay kit (Invitrogen A12216) according to the manufacturer’s instructions. Samples were pipetted into an opaque 96-well plate with a transparent bottom. Standards and samples were incubated with the Amplex Red reagent mixture at 37 °C for 30 min. For free cholesterol detection, cholesterol esterase (reagent G) was eliminated from the reagent mixture. Fluorescence was measured using excitation of 530–560 nm and emission detection at 590 nm. The level of cholesterol ester was calculation from the level of total cholesterol subtract to the level of free cholesterol. The levels of total cholesterol, free cholesterol, and cholesterol esters were normalized to the protein level of each individual sample.

### Immunohistochemistry of formalin fixed human brain tissue

The LBD brains were obtained from the Mayo Clinic Brain Bank for Neurodegenerative Disorders, which operates under protocols approved by the Mayo Clinic Institutional Review Board. Written informed consents were obtained by the Mayo Clinic Brain Bank for Neurodegenerative Disorders for brain autopsy and for the use of material and clinical data for research purposes, in compliance with institutional and national ethical guidelines. Brains were removed according to a rapid autopsy protocol. Specimens were fixed in 10% buffered formalin and processed for embedding in paraffin. In this study, only LBD cases with minimal Alzheimer type pathology (Braak stages 0–II and Thal phases 0–1) and known *APOE* genotype were included. Characteristics of the LBD cases are shown in Supplementary Table 2, online resource. Sections were deparaffinized, rehydrated and a heat-induced epitope retrieval was done in Tris–EDTA buffer (10 mM Tris base, 1 mM EDTA solution, 0.05% Tween20, pH-9.0) for 30 min at 95 °C followed by a 10 min rinse in cold water. Sections were washed four times in PBS between each incubation period. The endogenous peroxidase activity was quenched for 15 min in a mix of 3% hydrogen peroxide and 10% methanol in PBS. Sections were blocked for 1 h with blocking buffer containing 4% normal donkey serum, 2% BSA and 1 M glycine in PBS. Sections were then incubated with apoE antibody (Abcam, ab1907, 1:100) and pathogenic αSyn antibody NACP98 (a gift from Dr. Dickson, NACP98 specifically recognizes the C-terminus of αSyn at amino acid residue 98–115; 1:4000) in blocking buffer overnight at 4 °C. After washing three times with PBS, samples were incubated with fluorescently conjugated secondary antibodies for 2 h at room temperature and washed three times with PBS. Autofluorescence was eliminated with TrueBlack Lipofuscin Autofluorescence Quencher (Biotium, #23007) before mounting with the glass coverslip. Fluorescent signals were detected by Keyence fluorescence microscopy (model BZ-X, Keyence). Images with LB staining were captured for quantification. A total of 68 images and 125 LBs of non-*APOE4* group, 68 images and 139 LBs of *APOE4* group were captured at × 40 magnification and analyzed. The percentage of the αSyn-positive area that colocalizes with apoE was quantified using ImageJ software.

### Statistical analyses

For cerebral organoid comparisons between two groups, the Mann–Whitney *U* test was performed to determine the significance using GraphPad Prism. For rescue experiments and lipidomics analysis, One-way ANOVA and two-way ANOVA were performed to determine statistical significance using GraphPad Prism, respectively. Spearman correlation analysis was used to assess the association between proteins using GraphPad Prism. All statistical tests were two-sided. Data were presented as Mean ± SEM. A *p* value of < 0.05 was considered statistically significant. Specific statistical methods, the numerosity of the experiments, and the significance levels for each analysis are described in the legends of individual figures.

## Results

### ApoE deficiency leads to insoluble αSyn accumulation and synaptic loss in iPSC-derived cerebral organoids

**Fig. 1:**
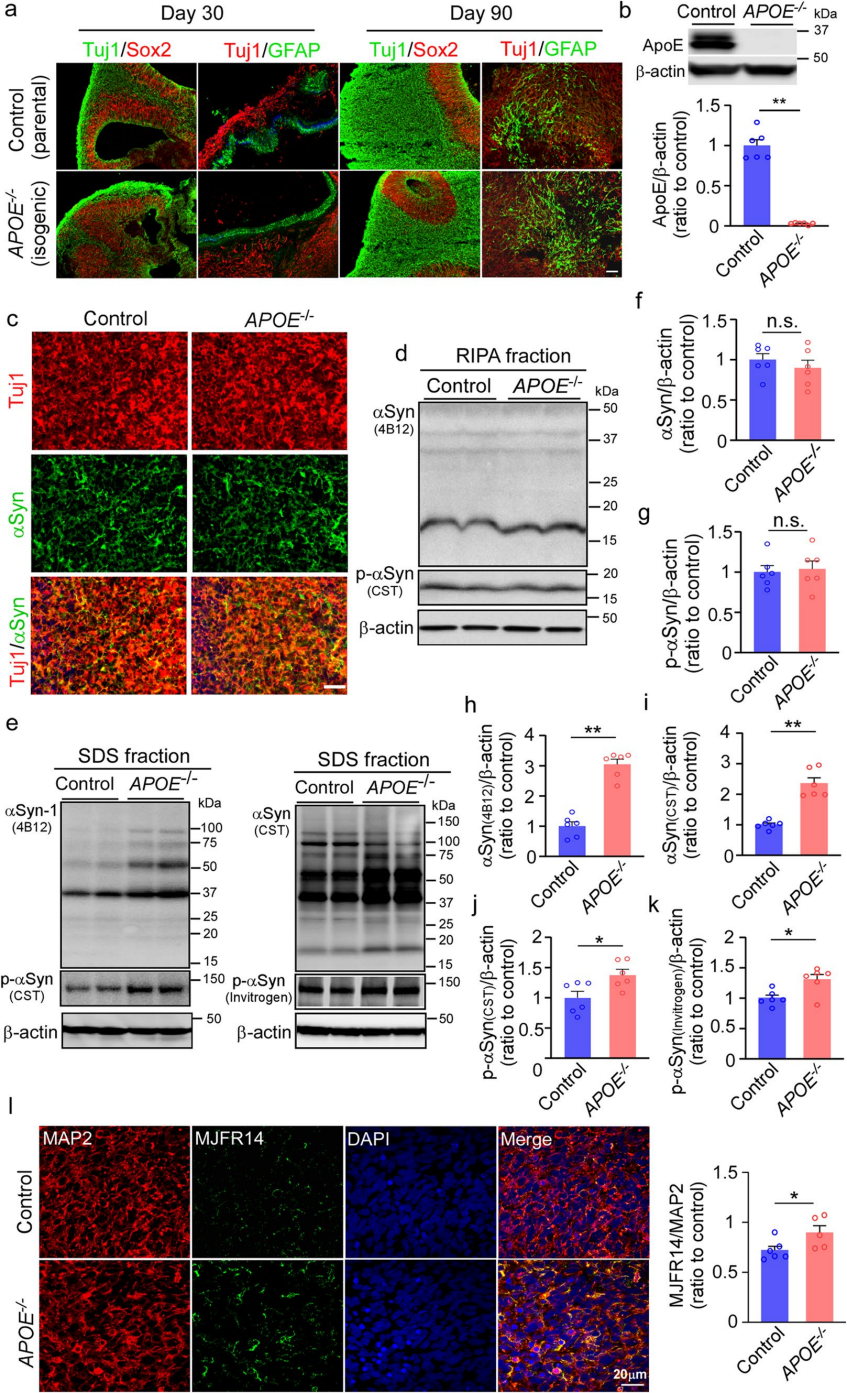
Exacerbated α-synuclein accumulation in apoE-deficient cerebral organoids. Parent control and isogenic *APOE* knockout (*APOE*^*−/−*^) iPSCs were differentiated into cerebral organoids. **a**, Representative images of the ventricular zone (VZ)-like structure (Tuj1, neuronal marker; SOX2, neural progenitor cell marker; and GFAP, astrocyte maker) in cerebral organoids at Day 30 and Day 90 of differentiation. Scale bar: 100 μm. **b**, ApoE depletion in isogenic *APOE*^*−/−*^ iPSC-derived cerebral organoids were confirmed by Western blotting at Day 90. **c**, Representative images of α-synuclein immunoreactivity in neurons of iPSC-derived cerebral organoids. Scale bar: 100 μm. **d–g**, Amounts of total α-synuclein (αSyn; **f**) and phosphorylated αSyn (p-αSyn; **g**) in the RIPA soluble fractions of cerebral organoids were quantified by Western blotting (**d**). **e**–**k**, Amounts of total αSyn (**e**, **h**, **i**) and p-αSyn (**e**, **j**, **k**) in the SDS-soluble fractions of cerebral organoids were quantified by Western blotting using two sets of different antibodies. ApoE, αSyn and p-αSyn levels were normalized to β-actin levels. 3 cerebral organoids were pooled and analyzed as one sample. All data are expressed as mean ± SEM (*n* = 6 samples/each). Experiments were repeated in three independent differentiation batches. **l**, Amounts of aggregated αSyn in cerebral organoids were quantified by MJFR14 (αSyn aggregate antibody) immunostaining. Scale bar: 20 μm. All data are expressed as mean ± SEM (*n* = 5–6 organoids/each). Mann–Whitney *U* tests were performed to determine statistical significance. *p < 0.05, **p < 0.01, n.s., not significant

We generated 3-D cerebral organoid models from a commercialized human parental iPSC line and its isogenic *APOE* knockout (*APOE*^*−/−*^) iPSC line from Xcell Science (Supplementary Table 1) using our previously reported protocol [71]. We confirmed that cerebral organoids from both parental and isogenic lines predominantly showed a dorsal forebrain region specification at Day 30, containing fluid-filled ventricular zone (VZ)-like structure aligned with Sox2-positive neural progenitors and beta-tubulin III (Tuj1)-positive neuroblasts in an outer layer (Fig. 1a). Immunostaining for glial fibrillary acidic protein (GFAP) showed the emergence of astrocytes with short processes in some VZ area at Day 30. At Day 90 of differentiation, the cerebral organoids showed a thicker Tuj1-positive outer layer, whereas GFAP-positive astrocytes increased in number and migrated within the neuronal layers, displaying typical mature astrocyte morphology with long processes (Fig. 1a). The expression of apoE was undetectable by Western blotting in the *APOE*^*−/−*^ iPSC-derived cerebral organoids (Fig. 1b). When the cerebral organoids were immunostained with αSyn antibody (4B12), αSyn was abundantly detected in Tuj1-positive neurons in the cerebral organoids from both iPSC lines at Day 90 (Fig. 1c). Total αSyn and phosphorylated αSyn (p-αSyn) in the cerebral organoids in both RIPA buffer and SDS buffers were quantified (Fig. 1d-k). We found that the amounts of αSyn and p-αSyn significantly increased in the detergent-insoluble SDS fraction of *APOE*^*−/−*^ iPSC-derived cerebral organoids compared to those from the parent controls (Fig. 1h-k), although no significant differences were detected in the detergent soluble RIPA fraction (Fig. 1f-g). Immunostaining of aggregated αSyn using MJFR14 antibody also confirmed the significant increase of αSyn aggregates in *APOE*^*−/−*^ cerebral organoids (Fig. 1l). To investigate a potential involvement of Aβ pathology in αSyn accumulation in *APOE*^*−/−*^ cerebral organoids, we quantified the amount of Aβ40 and Aβ42 in both RIPA and SDS fractions by ELISA. In contrast to the increase of insoluble αSyn accumulation, Aβ42 levels showed a significant decrease in the SDS fraction of *APOE*^*−/−*^ cerebral organoids (Supplementary Fig. 2j-m, online resource). We further investigated the effects of apoE deficiency on the neuronal synaptic proteins and found significant reductions of presynaptic synaptophysin and postsynaptic PSD95 protein levels in the *APOE*^*−/−*^ cerebral organoids compared to controls at Day 90. The levels of an apoptotic marker cleaved caspase 3 and the size of cerebral organoids did not change in the *APOE*^*−/−*^ cerebral organoids (Supplementary Fig. 2, online resource). Consistent with the Western blotting results, immunostaining also found a trend of decrease in the pre-synaptic marker (Synaptophysin) and post-synaptic marker (PSD95) in the *APOE*^*−/−*^ cerebral organoids compared to controls at Day 90 (Supplementary Fig. 2e-g, online resource). Together, these results indicate that apoE deletion promotes the accumulation of insoluble αSyn and influences synaptic hemostasis in the cerebral organoids, which is independent of Aβ pathology.

### ApoE deficiency impacts the transcriptional profiles in iPSC-derived cerebral organoids

**Fig. 2:**
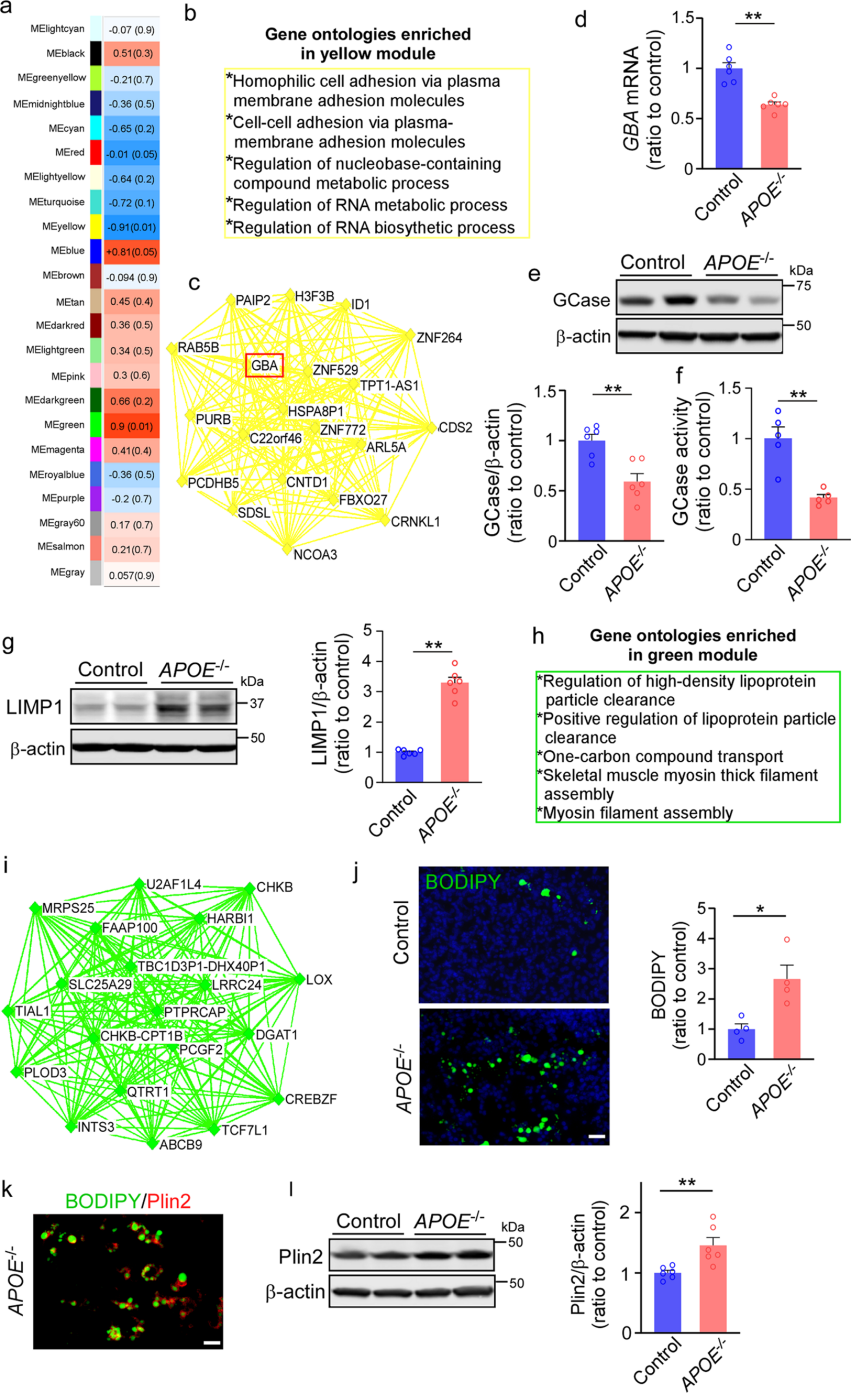
Altered transcriptional profiles of apoE-deficient cerebral organoids implicating GBA and lipid-related pathways. RNA-seq was performed on parental control and isogenic *APOE*^*−/−*^ iPSC-derived cerebral organoids at Day 90 (3 cerebral organoids were pooled and analyzed as one sample, *n* = 3 samples/each). **a**, Module-trait relationships between groups revealed by WGCNA are shown. The correlation coefficient (r) and the correlation *p*-value in the parentheses are indicated in each module. Orange indicates upregulation in *APOE*^*−/−*^ organoids; blue indicates downregulation in *APOE*^*−/−*^ organoids (upregulation in controls) **b**, Top gene ontologies enriched by the yellow module genes. **c**, Interaction of top 20 genes with the highest connectivity among each other in the yellow module. **d–e**, GBA mRNA expression (**d**) and β-glucocerebrosidase (GCase) levels in the RIPA lysates (**e**) were quantified by RT-qPCR and Western blotting at Day 90, respectively. **f**, GCase activity in cerebral organoids was detected by GCase activity kit (Fluorometric). Data were normalized to protein concentrations. **g**, Amounts of LIMP1 in the iPSC-derived cerebral organoids at Day 90 were quantified by Western blotting. **h**, Top gene ontologies enriched by the green module genes. (**i**) Interaction of top 20 genes with the highest connectivity among each other in the green module. **j**, The lipid droplet accumulation was evaluated by BODIPY staining (Scale bar: 20 μm) with the fluorescent intensity quantified by Image J. All data are expressed as mean ± SEM (*n* = 4 organoids/each). **k**, Representative images of co-staining of BODIPY and Plin2 (lipid droplet membrane marker). Scale bar: 20 μm. **l**, Amounts of Plin2 in the iPSC-derived cerebral organoids at Day 90 were quantified by Western blotting. 3 cerebral organoids were pooled and analyzed as one sample. All data are expressed as mean ± SEM (*n* = 6 samples/each). Experiments were repeated in three independent differentiation batches. Mann–Whitney U tests were performed to determine statistical significance. *p < 0.05, **p < 0.01

Next, we performed RNA-sequencing (RNA-seq) on the iPSC-derived cerebral organoids at Day 90. Principal component analysis (PCA) demonstrated clear separation between parental and isogenic *APOE*^*−/−*^ cerebral organoids (Supplementary Fig. 3a, online resource). We identified 2307 differentially expressed genes (DEGs) between control (Con) and *APOE*^*−/−*^ cerebral organoids. Numbers of DEGs assigned to neurons and astrocytes defined through CIBERSORT and CellCODE program were 1297 and 1010, respectively. No significant differences in neuron and astrocyte proportion were observed between groups (Supplementary Fig. 3b–d, online resource). Weighted Gene Co-expression Network Analysis (WGCNA) identified two gene modules that were significantly different between *APOE*^*−/−*^ and controls (yellow and green, *p* = 0.01) and two gene modules that were marginally significant (red and blue, *p* = 0.05) (Fig. 2a). The yellow module was downregulated in *APOE*^*−/−*^ cerebral organoids compared to controls. Genes in the module were enriched in gene ontologies (GO) related to plasma membrane and RNA metabolism (Fig. 2b). Interestingly, *GBA* was identified as one of the top hub genes in the yellow module (Fig. 2c). *GBA* gene encoding the lysosomal enzyme β-glucocerebrosidase (GCase) is the most common known genetic risk factors for the development of Parkinson’s disease and related synucleinopathies [12, 19, 57]. We found that apoE deficiency results in the reduction of *GBA* expression at the mRNA and protein levels in the cerebral organoids detected by RT-qPCR and Western blotting, respectively (Fig. 2d, e). Consistently, GCase activity assay showed a significant decrease in the enzyme activity of GCase in *APOE*^*−/−*^ cerebral organoids compared to the controls (Fig. 2f). We found that apoE deficiency also increased the level of CD63/LIMP1, which is a main component of the lysosomal membrane [56] (Fig. 2g). The green module was upregulated in *APOE*^*−/−*^ cerebral organoids. Genes in the module were enriched in GO processes related to the regulation of HDL particles clearance (Fig. 2h). Indeed, several hub genes of the green module were involved in the pathways for lipid transport (*ABCB9*) [53] and lipid droplet formation (*LOX, DGAT1*) [9, 46] (Fig. 2i). Thus, to examine the effect of apoE deficiency on lipid metabolism and storage, we stained lipid droplet in cerebral organoids with BODIPY, a dye that specifically labels neutral lipids and is commonly used to detect lipid droplets [23, 38]. We found greater BODIPY-positive lipid droplet accumulation in *APOE*^*−/−*^ cerebral organoids compared to controls (Fig. 2j). Immunostaining showed that Perilipin 2 (Plin2) is a surface protein of lipid droplet (Fig. 2k). Western blotting also confirmed a significant increase of Plin2 level in the *APOE*^*−/−*^ cerebral organoids (Fig. 2l). The red module, enriched for cytoplasmic transport and intracellular membrane-bounded organelle was downregulated in the *APOE*^*−/−*^ cerebral organoids (Supplementary Fig. 3f, online resource), which included *NUP98*, *LCORL*, *POLR3D*, *MAGEL2*, *VSIG10*, and *MRPL17* as the top hub genes (Supplementary Fig. 3 g, online resource). Consistent with the results from WGCNA, *VPS39* was the most significantly altered gene among all DEGs (Supplementary Fig. 3e, online resource). Vps39 is one of the subunits of the homotypic fusion and vacuole protein sorting (HOPS) tethering complex required for the clustering and fusion of late endosomes and lysosomes [50]. The protein level of Vps39 was also decreased in *APOE*^*−/−*^ cerebral organoids when analyzed by Western blotting, whereas the expression of endosome marker EEA1 and lysosome marker LAMP1 were increased (Supplementary Fig. 3 h–k, online resource). No significant change was observed in the level of endoplasmic reticulum marker calnexin between groups (Supplementary Fig. 3 l, online resource) suggesting a selective alteration of the endocytic trafficking. Together, these results suggest an important role of apoE in GCase regulation, lipid droplet formation and endo-lysosomal trafficking in the iPSC-derived cerebral organoids.

### ApoE deficiency alters the lipid profiles in iPSC-derived cerebral organoids

**Fig. 3:**
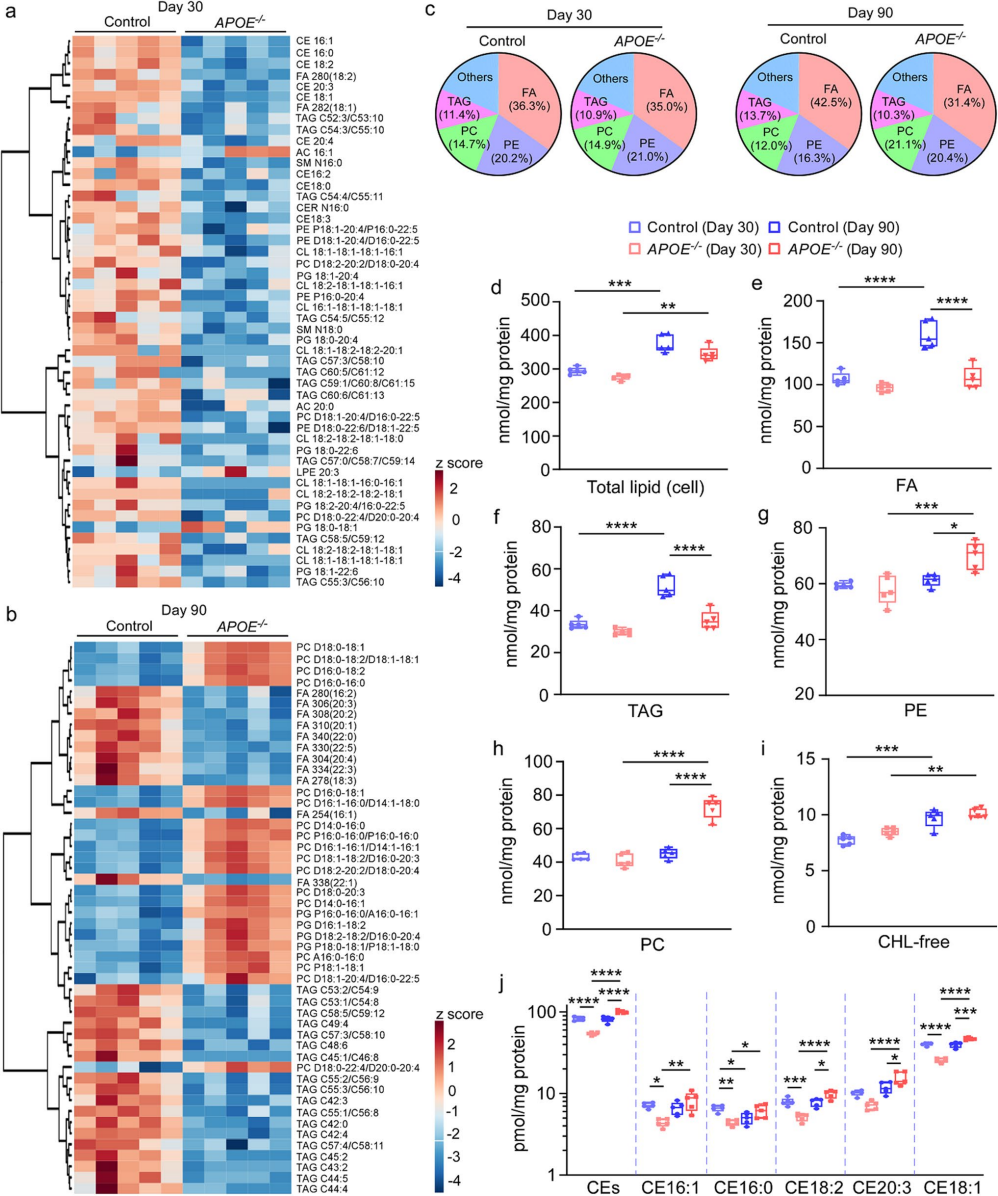
Impaired lipid profiles in apoE-deficient cerebral organoids. Lipidomics and cholesterol assay were performed on lysates from parental control and isogenic *APOE*^*−/−*^ iPSC-derived cerebral organoids at Day 30 and Day 90. **a–b**, Heatmaps of top 50 lipid species significantly altered by apoE-deficiency at Day 30 (**a**) and Day 90 (**b**) are shown. **c**, Overall composition of lipid species in the lysates of iPSC-derived cerebral organoids. **d–j**, Concentration of total lipid (**d**), fatty acyl chains in triacylglycerol (FA, **e**), triacylglycerol (TAG, **f**), phosphatidylethanolamine (PE, **g**), phosphatidylcholine (PC, **h**), free cholesterol (CHL-free, **i**) and cholesterol ester (CE) species (**j**) in the lysates were plotted. All lipid concentrations were normalized to the protein levels. Lysates and culture media from 3 cerebral organoids were analyzed as one sample. All data are expressed as mean ± SEM (*n* = 5 samples/each). Two-way ANOVA were performed to determine statistical significance. *p < 0.05, **p < 0.01, *** *p* < 0.001, **** *p* < 0.0001

To further explore the effects of apoE deficiency on the lipid metabolism, lipidomics was conducted through shotgun lipidomics analysis in both cell lysates (Fig. 3, Supplementary Fig. 4, online resource) and conditioned medium (Supplementary Fig. 5, online resource) from the iPSC-derived cerebral organoids at Day 30 and Day 90. The lipidomes in the lysates from *APOE*^*−/−*^ cerebral organoids were broadly altered compared to those of the controls (Fig. 3a, b). Lipidomics identified four main lipid species in the cerebral organoids: fatty acyl chains in triacylglycerol (FA), phosphatidylethanolamine (PE), phosphatidylcholine (PC), and triacylglycerol (TAG). Although the ratios of these main lipid species were similar in both groups at Day 30 (Control: FA 36.3%, PE 20.2%, PC 14.7%, TAG 11.4%; *APOE*^*−/−*^: FA 35%, PE 21%, PC 14.9%, TAG 10.9%), the lipid composition changed at day 90 (Control: FA 42.5%, PE 16.3%, PC 12%, TAG 13.7%; *APOE*^*−/−*^: FA 31.4%, PE 20.4%, PC 21.1%, TAG 10.3%) (Fig. 3c). Total lipid levels were increased during maturation of iPSC-derived organoids as they were significantly higher at Day 90 than those at Day 30 regardless of apoE deficiency (Fig. 3d). The levels of FA and TAG decreased in the *APOE*^*−/−*^ cerebral organoids at Day 90, whereas the levels of PE and PC increased (Fig. 3e–h). We also found that apoE deficiency reduced ceramide (CER) but increased sphingomyelin (SM) in the cerebral organoids at Day 90 without evident influences on other lipid species such as free cholesterol (CHL-free, Fig. 3i), cardiolipin (CL), lyso-cardiolipin (LCL), phosphatidic acid (PA), phosphatidylglycerol (PG), phosphatidylinositol (PI), phosphatidylserine (PS), lyso-phosphatidylethanolamine (LPE), acylcarnitine (AC), and lyso-phosphatidylcholine (LPC) (Supplementary Fig. 4, online resource). Although the levels of cholesterol esters (CEs) detected through cholesterol assay in the lysates of the *APOE*^*−/−*^ cerebral organoids were lower than those from the control at Day 30, apoE deficiency rather increased CEs in the cerebral organoids at Day 90 (Fig. 3j). When lipidomics in the culture medium was performed, there were much less detectable lipidomes compared to those in the lysates although the decreased levels of LPC were detected in the culture medium from the *APOE*^*−/−*^ cerebral organoids at Day 30 (Supplementary Fig. 5f, online resource). While the levels of CHL-free (Supplementary Fig. 5d, online resource) and CEs (Supplementary Fig. 5j, online resource) in the medium decreased along with the maturation of iPSC-derived cerebral organoids, CHL-free levels were significantly decreased in the medium from *APOE*^*−/−*^ cerebral organoids at Day 90. Taken together, these results suggest that apoE deficiency causes broad changes in the metabolism of lipids, particularly cholesterol, in the iPSC-derived cerebral organoids at different maturation stages.

### Confirmation of phenotypic features in a second independent *APOE*^*−/−*^ isogenic iPSC line

To evaluate if the findings obtained from one *APOE*^*−/−*^ isogenic line could be reproduced using a second and independently generated genome-engineered line, we generated another set of *APOE*^*−/−*^ isogenic line using Clustered Regularly Interspaced Short Palindromic Repeats (CRISPR)-Cas9 technology. Cerebral organoids were generated from both the parental and isogenic lines; the expression of neuronal marker Tuj1 and astrocyte marker GFAP were confirmed at Day 90 (Supplementary Fig. 6a, online resource). *APOE* depletion efficiency in the isogenic line was confirmed by detecting the *APOE* mRNA and protein levels in cerebral organoids at Day 90 (Supplementary Fig. 6b–d, online resource). This independent mutant line reproduced the features displayed by the first *APOE*^*−/−*^ line, including dysregulation of intracellular organelles, synaptic loss, lipid droplet accumulation, and reduction of GBA levels and activity, accompanied with αSyn and p-αSyn accumulation in the detergent insoluble fraction (Supplementary Fig. 6c, e–n; Supplementary Fig. 7, online resource).

### Exogenous astrocytic apoE2 and apoE3, but not apoE4, alleviate pathological phenotypes in *APOE*^*−/−*^ iPSC-derived cerebral organoids

**Fig. 4:**
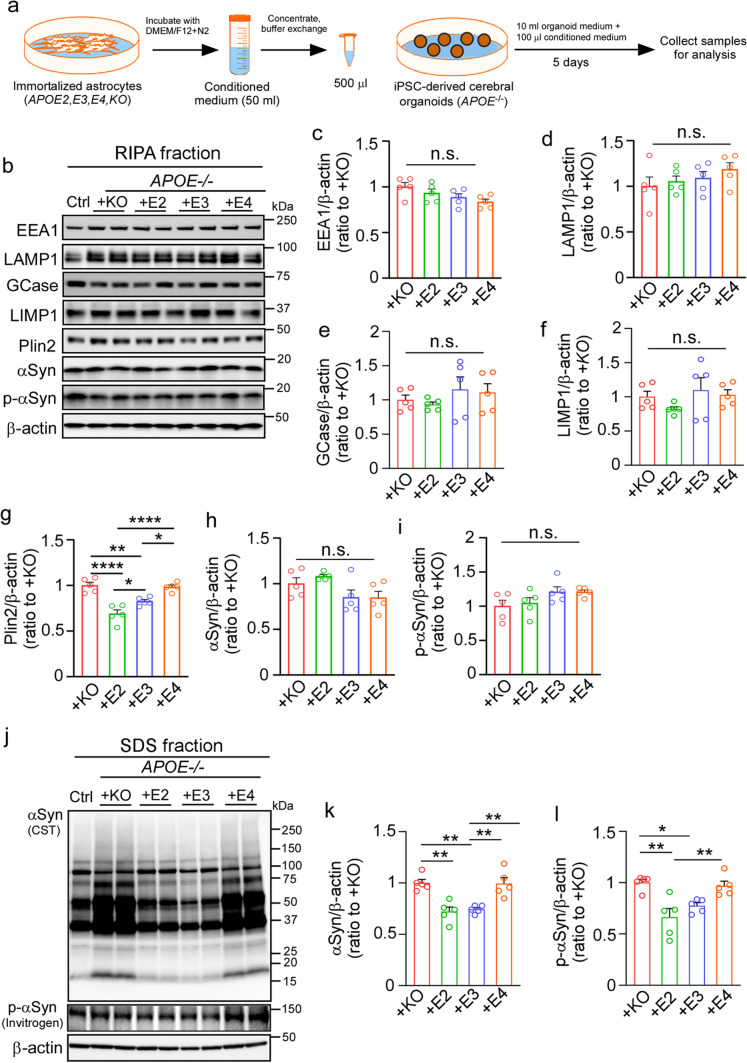
ApoE2 and apoE3, but not apoE4, partially rescue α-synuclein accumulation in apoE-deficient cerebral organoids. The *APOE*^*−/−*^ iPSC-derived cerebral organoids at Day 90 were treated with conditioned media of immortalized astrocytes from *APOE2*-target replacement (TR), *APOE3*-TR*,* or *APOE4*-TR mice for 5 days. Conditioned media from *Apoe-*KO astrocytes were used as a control. **a**, A schematic workflow of the rescue experiments. **b–i**, Amounts of EEA1 (**c**), LAMP1 (**d**), GCase (**e**), LIMP1 (**f**), Plin2 (**g**), αSyn (**h**) and p-αSyn (**i**) in the RIPA fraction of the cerebral organoids after treatments were quantified by Western blotting. **j–l**, Amounts of total αSyn (**k**) and p-αSyn (**l**) in the SDS fraction of cerebral organoids after treatments were quantified by Western blotting. All data were normalized to β-actin levels. 3 cerebral organoids were pooled and analyzed as one sample. All data are expressed as mean ± SEM (*n* = 5 samples/each). Experiments were repeated in three independent differentiation batches. One-way ANOVA was performed to determine statistical significance. **p < 0.01, ****p < 0.0001, n.s., not significant

To determine whether the pathological phenotypes induced by apoE deficiency can be rescued by reintroducing different exogenous apoE isoforms, we treated the *APOE*^*−/−*^ cerebral organoids with conditioned media from immortalized astrocytes derived from *Apoe*-knockout, *APOE2*-target replacement (TR), *APOE3*-TR*,* or *APOE4*-TR mice for 5 days (Fig. 4a). Western blotting showed that the addition of exogenous astrocytic media containing apoE2 and apoE3 isoforms reduced the level of Plin2 in the *APOE*^*−/−*^ cerebral organoids compared to the media from *Apoe*-KO astrocytes or containing apoE4, although there was no effect on the levels of EEA1, LAMP1, GCase, LIMP1, αSyn and p-αSyn in the RIPA fraction (Fig. 4b–i). No significant differences were detected in the total cholesterol, free cholesterol, and CE levels (Supplementary Fig. 8a–c, online resource). In addition, the amounts of insoluble αSyn and p-αSyn in the SDS fraction were significantly decreased in the *APOE*^*−/−*^ cerebral organoids when treated with exogenous astrocytic media containing apoE2 and apoE3, but not apoE4, compared to those from *Apoe*-knockout astrocytes (Fig. 4j–l, Supplementary Fig. 8d–f, online resource). These results indicate that astrocyte-derived apoE2 and apoE3, but not apoE4, are capable of alleviating αSyn accumulation and lipid droplet formation in the *APOE*^*−/−*^ iPSC-derived cerebral organoids.

### *APOE4* increases αSyn accumulation in the iPSC-derived cerebral organoids and human brains

**Fig. 5:**
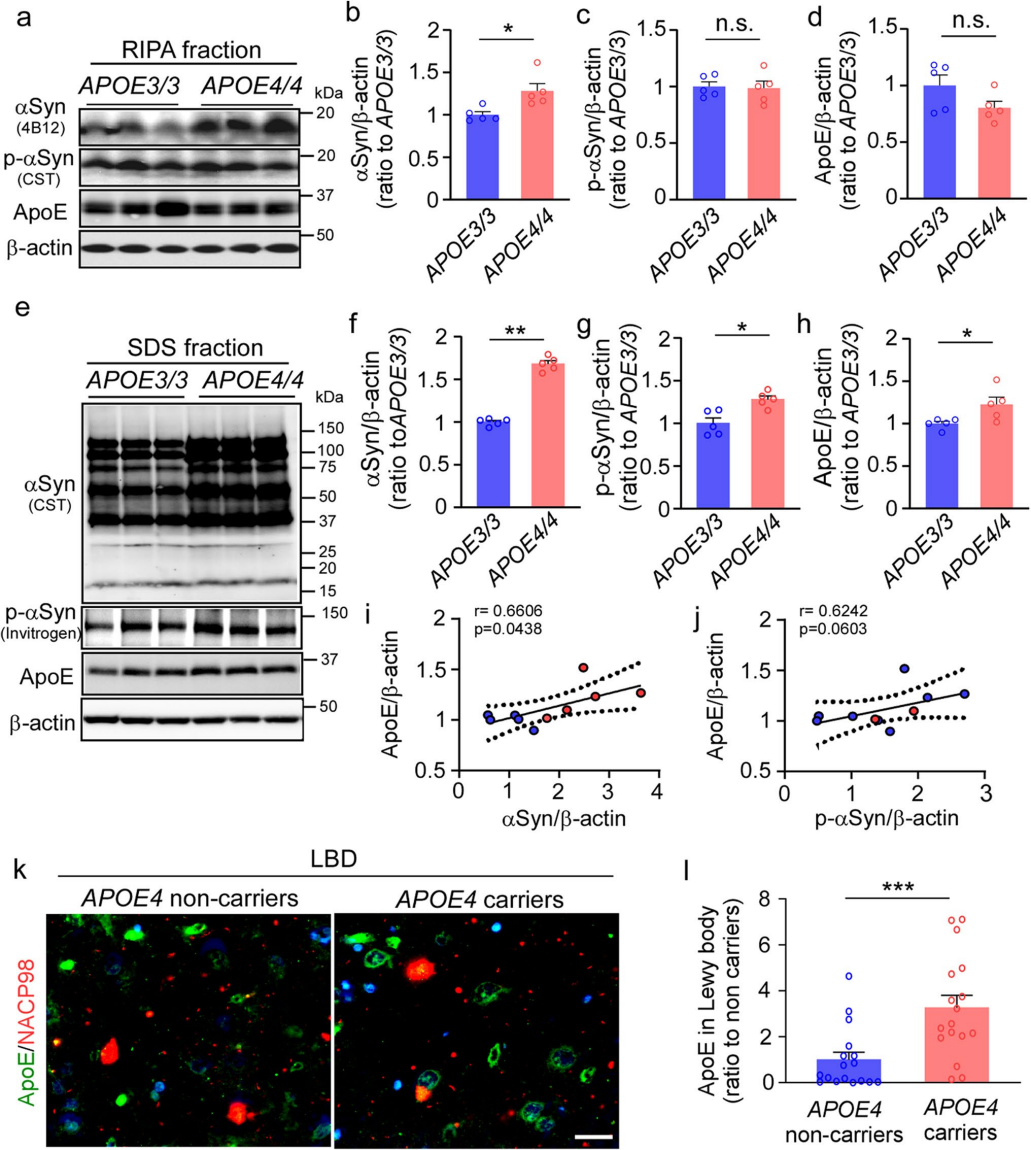
Exacerbated α-synuclein accumulation in apoE4 cerebral organoids and postmortem brains from *APOE4* carriers. Cerebral organoids were generated from iPSC lines carrying *APOE* ε3/ε3 (*APOE3/3*) or ε4/ε4 (*APOE4/4*) genotype and subjected to analyses at Day 90. **a–d**, Amounts of αSyn (**b**), p-αSyn (**c**) and apoE (**d**) in the RIPA fraction of the iPSC-derived cerebral organoids were quantified by Western blotting. **e–j**, Amounts of αSyn (**f**), p-αSyn (**g**) and apoE (**h**) in the SDS fraction of the iPSC-derived cerebral organoids were quantified by Western blotting. ApoE, αSyn, and p-αSyn levels were normalized to β-actin levels. Spearman correlation analyses for apoE vs. αSyn (**i**) and apoE vs. p-αSyn (**j**) in SDS fraction are shown with the correlation coefficient (*r*) and the correlation *p* value. Experiments were repeated in two independent differentiation batches. Lysates of 3 cerebral organoids from each line were analyzed as one sample. All data are expressed as mean ± SEM (*N* = 5 lines/each). **k–l**, Postmortem brain sections from the superior temporal cortex of Lowy body dementia (LBD) subjects with or without *APOE4* were immunostained with apoE antibody and anti-αSyn NACP98 antibody for Lewy body. Representative images for the deposition of apoE in NACP-positive Lewy bodies are shown (**k**). Scale bar: 20 µm. The colocalization of apoE with NACP-positive Lewy bodies was quantified by Image J (**l**). All data are expressed as mean ± SEM (*N* = 17 patients/each). Mann–Whitney *U* tests were performed to determine statistical significance. *p < 0.05, **p < 0.01, ***p < 0.001

We generated cerebral organoids using iPSC lines from cognitively unimpaired individuals carrying homozygous *APOE3* (*N* = 5) or *APOE4* (*N* = 5) collected from multiple sources (Supplementary Table 1, online resource). To focus on the comparison between apoE3 and apoE4 isoform in the cerebral organoids, heterozygous *APOE3/E4* lines were not included in this experiment. Consistent with the results from exogenous astrocytic apoE, we found a significant increase of BODIPY-positive lipid droplets as well as Plin2 and CE levels in the cerebral organoids with homozygous *APOE4* compared to those with homozygous *APOE3* (Supplementary Fig. 9, online resource). When the levels of αSyn and p-αSyn in the RIPA fraction were measured by Western blotting at Day 90, we found that cerebral organoids with homozygous *APOE4* showed higher levels of αSyn than those with homozygous *APOE3* although no differences were detected in p-αSyn and apoE levels (Fig. 5a–d). The amounts of αSyn, p-αSyn and apoE in the SDS fraction were all significantly increased in the *APOE4* cerebral organoids (Fig. 5e–h, Supplementary Fig. 9, online resource). Interestingly, there is a trend of positive correlation between apoE and αSyn levels in SDS fraction of the cerebral organoids (Fig. 5i–j).

To further address relevance in human brains, we assessed the association of apoE and αSyn pathology in postmortem brain samples from Lewy body disease patients with minimal amyloid pathology (Supplementary Table 2, online resource). When the brain sections were immunostained with αSyn (NACP98) and apoE antibodies, apoE was detected in NACP98-positive Lewy bodies (Fig. 5k, Supplementary Fig. 10, online resource). Consistent with the results from the iPSC-derived cerebral organoids, we found significant increase of apoE deposition in Lewy bodies in *APOE4* carriers compared to those of age- and sex-matched non-carriers (Fig. 5l). These findings suggest that apoE4 facilitates αSyn accumulation by modulating lipid metabolism and/or through their direct interaction with αSyn-containing Lewy bodies.

## Discussion

Deficiency of apoE in humans is rare, thus human-based experimental model systems are needed to assess the physiological and pathological roles of apoE, as well as the underlying mechanisms. In this study, using a human iPSC isogenic line deficient of apoE and the iPSC-derived cerebral organoids, we show that apoE expression is critical for maintaining lipid homeostasis and related membrane trafficking. More importantly, we show that apoE is critical for preventing αSyn aggregation and deposition wherein we also demonstrate apoE isoform-dependent effects in rescuing the phenotypic outcomes. Together with validation using human postmortem brains, our work demonstrates critical roles of apoE in brain homeostasis and offers critical insights into why *APOE* is a strong genetic risk factor for multiple neurodegenerative diseases including synucleinopathies.

A major physiological function of αSyn is to maintain a synaptic vesicle reserve pool within the presynaptic terminal through its lipid-binding domain [34, 43]. However, under pathological conditions, the interaction of αSyn with cellular lipid membrane might contribute to the conformational changes of αSyn from its physiological soluble form to toxic insoluble aggregates [38]. Therefore, a possible involvement of lipid metabolism pathway has been increasingly recognized in the pathogenesis of Lewy body diseases [2, 59]. While apoE is the most abundant apolipoprotein in the brain [68], our iPSC-derived cerebral organoid models demonstrate that *APOE* deficiency results in increased accumulation of insoluble αSyn and p-αSyn as well as altered lipid profiles and excess lipid droplet formation, events that are confirmed using another independent iPSC line. The formation of lipid droplets, composed of neutral lipids such as TAG and CE, is induced through various environmental and cellular conditions, including elevated concentrations of extracellular lipids, inflammatory events, increased ROS levels and intracellular metabolic changes [5, 18, 25, 52]. Because excess lipid droplets might represent abnormal cellular lipid homeostasis, it is predicted that the altered lipid environment facilitates the phosphorylation and pathological aggregation of αSyn. Multiple studies also show that the association of αSyn with lipid membrane promotes its aggregation via facilitating a focal αSyn accumulation and/or inducing the conformational change into “seed” and/or aggregation-prone oligomers [59]. Under normal condition, αSyn adopts an α-helical conformation when bound to membranes. The helical structure is important in prevention of β-sheet formation, which leads to further α-Syn fibrillization [13, 24, 43]. ApoE deficiency likely modifies the membrane composition and influences the membrane binding of α-Syn, which might induce the β-sheet formation and α-Syn aggregation. It is also possible that apoE suppresses αSyn aggregation by directly interfering the interaction between αSyn and cellular membranes. As overexpression of the pathogenic αSyn A53T causatively increases the lipid droplet accumulation in dopaminergic neurons [55], it is tempting to speculate that apoE loss of function may trigger the vicious cycle between abnormal lipid droplet accumulation and αSyn pathology in Lewy body diseases. Since the proper regulation of lipid metabolism is critical for maintaining synaptic homeostasis [48], apoE loss of function and αSyn accumulation may synergistically accelerate synapse loss by affecting synaptic lipid distribution beyond αSyn toxicity.

Intriguingly, our results demonstrate lower levels of *GBA* in the *APOE*^*−/−*^ cerebral organoids. *GBA* is a major genetic risk factor for neurodegenerative diseases with synucleinopathies such as PD and DLB [19, 57]. GCase coded by *GBA* gene mediates the degradation of glucosylceramide into glucose and ceramide in lysosomes [6, 57]. Consistently, lipidomics analysis reveals the decrease of ceramide level in the lysates of *APOE*^*−/−*^ cerebral organoids. While *GBA* loss of function variants and suppression of GCase expression have been shown to increase αSyn accumulation [40, 65], pharmacological activation of GCase ameliorates pathological accumulation of αSyn in iPSC-derived dopaminergic neurons [41]. Glucosylceramide likely promotes the oligomerization of αSyn in acidic condition (pH 5.0) [40]. Thus, the excess accumulation of insoluble αSyn in the *APOE*^*−/−*^ cerebral organoids may be partially due to the reduction of *GBA* expression, although further studies are necessary to define the functional link between *APOE* and *GBA*. In addition to lipid metabolism, WGCNA also identifies multiple modules affected by apoE deficiency, which include pathways related to plasma membrane metabolism and intracellular organelle transport. Supporting this, we find a significant decrease of Vps39 which mediates the fusion of late endosomes and lysosomes [50], as well as increased levels of endo-lysosomal membrane markers in the *APOE*^*−/−*^ cerebral organoids. Thus, altered membrane lipid distribution due to apoE deficiency may cause endo-lysosomal dysregulation, which might contribute to lipid droplet accumulation and *GBA* loss of function.

Of note, increasing lines of evidence indicate that *APOE4* exacerbates αSyn pathology and related toxicity independent of amyloid [8, 72]. Consistently, we also observe the increased levels of αSyn and p-αSyn in the insoluble fractions of cerebral organoids carrying homozygous *APOE4* compared to those with homozygous *APOE3*. Furthermore, key phenotypes observed in apoE-deficient cerebral organoids including the lipid droplet accumulation are recapitulated in *APOE4* cerebral organoids, suggesting that a possible pathogenic pathway related to *APOE4* is its decreased ability to mediate lipid metabolism. Indeed, disturbances of lipid homeostasis have been reported as a common event in neurodegenerative diseases [15]. Thus, *APOE4*-associated dysregulation of cholesterol homeostasis likely contributes to the accelerated accumulation of insoluble αSyn in cerebral organoids. Importantly, we find that exogenous astrocytic apoE ameliorates the pathogenic phenotypes related to lipid metabolism and αSyn accumulation in the *APOE*^*−/−*^ cerebral organoids in an isoform-dependent manner, again suggesting that deleterious *APOE4* effect on αSyn accumulation is mediated through its loss of function property.

It is interesting to note that *APOE4* knockin mice display more p-αSyn levels compared to *Apoe*-KO mice upon breeding to the A53T mutant αSyn transgenic mouse model [8]. Further, apoE deficiency increases αSyn solubility in A30P mutant αSyn transgenic mice [16]. Thus, when αSyn carries pathological mutations or is present in pathogenic insoluble forms, apoE may rather exacerbate αSyn aggregation through direct physical interaction [13, 14]. Supporting this, we find the positive correlation between apoE and αSyn in the insoluble SDS fraction of cerebral organoids, where *APOE4* cerebral organoids have higher insoluble apoE levels than *APOE3* cerebral organoids. Furthermore, greater amount of apoE is colocalized with Lewy bodies in human postmortem brains from *APOE4* carriers compared to those from non-carriers. Thus, apoE and αSyn likely co-aggregate, a process that is likely accelerated in the presence of apoE4. More studies are needed to define if the co-deposition of apoE and αSyn is causatively or consequently involved in the development of Lewy bodies. In contrast to the increase of αSyn pathology, insoluble Aβ42 levels in SDS fraction rather decreased in the apoE-deficient cerebral organoids. As the amount of Aβ42 does not differ in the iPSC lines from normal individuals with different *APOE* genotypes as reported in our previous study [71], the effects of *APOE4* on αSyn pathology are likely independent of Aβ pathology in the cerebral organoids. Nonetheless, since it has been reported that Aβ plaques could promote seeding and spreading of αSyn in a mouse model of Lewy body dementia with Aβ pathology [4], further studies are needed to investigate how *APOE* and Aβ impact αSyn pathology under disease conditions.

In summary, our study demonstrates that apoE deficiency induces the accumulation of insoluble αSyn and p-αSyn accompanied with increased synapse loss in 3-D human iPSC-derived cerebral organoids. ApoE deficiency is also associated with the excess lipid droplet formation, *GBA* reduction and endo-lysosomal dysregulation likely due to abnormal lipid metabolism. While *APOE4* cerebral organoids have higher levels of insoluble αSyn than *APOE3* cerebral organoids, *APOE4* may mediate these pathological outcomes through both loss of physiological function and gain of pathological function mechanisms. These findings provide novel insights into the differential effects of apoE isoforms on synucleinopathies, highlighting the critical roles of lipid metabolism and membrane trafficking. Since apoE is also produced by microglia and vascular mural cells [68], our future studies will utilize iPSC-derived cerebral organoid systems with the incorporation of additional brain cell types. Together, these studies will address the underlying pathogenic mechanisms of synucleinopathies in human relevant models, providing guidance on apoE-targeted, mechanism-based therapy.

## Supplementary Information

Below is the link to the electronic supplementary material.

Supplementary file1 (PDF 4174 KB)

## Data Availability

All relevant data are available from the corresponding author upon reasonable request. The RNA-seq and lipidomics data are available via the AD Knowledge Portal [https://adknowledgeportal.synapse.org]. The AD Knowledge Portal is a platform for accessing data, analyses, and tools generated by the Accelerating Medicines Partnership (AMPAD) Target Discovery Program and other National Institute on Aging (NIA)-supported programs to enable open-science practices and accelerate translational learning. The data, analyses, and tools are shared early in the research cycle without a publication embargo on secondary use. Data are available for general research use according to the following requirements for data access and data attribution [https://adknowledgeportal.synapse.org/DataAccess/Instructions]. For access to content described in this manuscript see [https://www.synapse.org/#!Synapse:syn25914210].
