# Supplementary material for: Apolipoprotein E regulates lipid metabolism and α-synuclein pathology in human iPSC-derived cerebral organoids
Source: Acta Neuropathol. 2021 Aug 28;142(5):807–25. doi: 10.1007/s00401-021-02361-9 (PMC8500881; doi:10.1007/s00401-021-02361-9)
Supplement: Supplementary file 1 — Supplementary file1 (PDF 4174 KB) [file 401_2021_2361_MOESM1_ESM.pdf]

Supplementary information

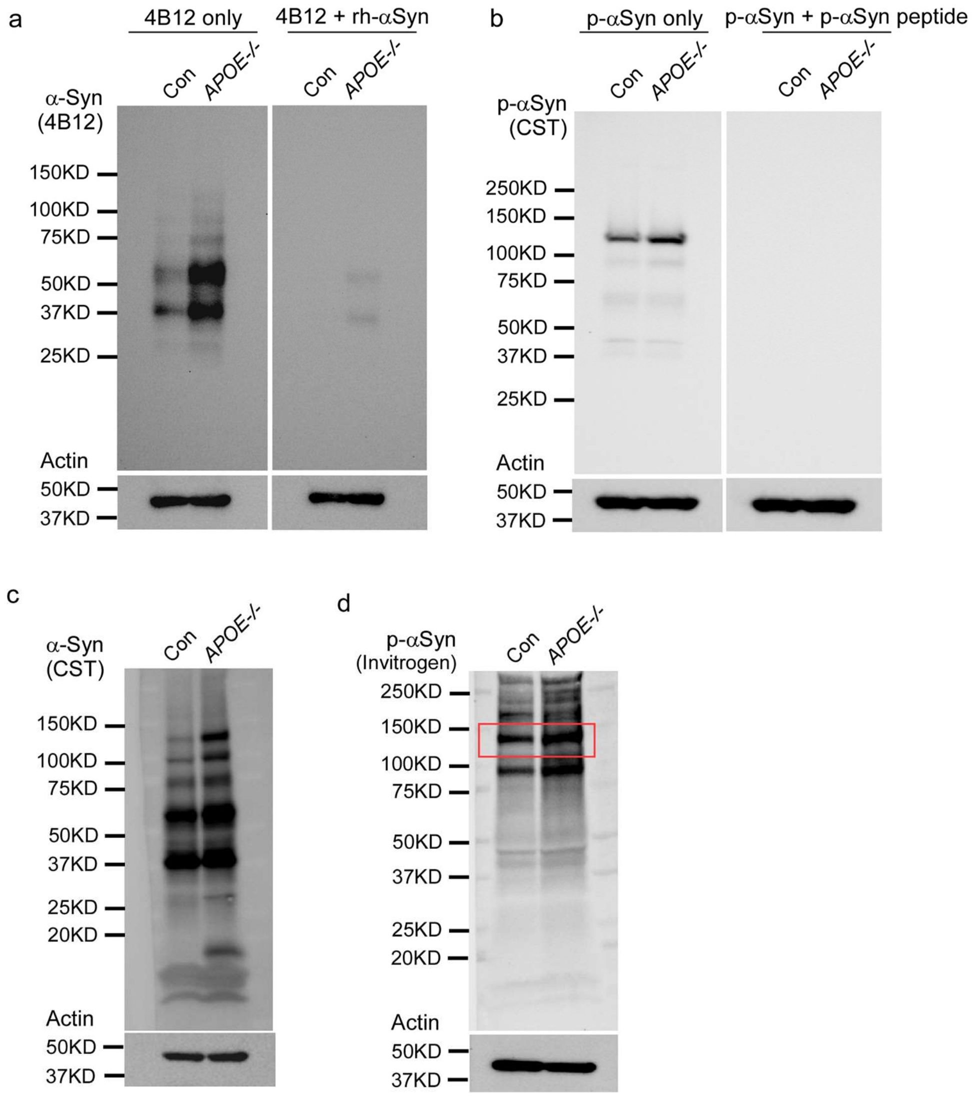

Supplementary Figure 1. Validation of αSyn and p-αSyn antibodies.
a-b, To validate the specificity of αSyn and p-αSyn antibodies, the antibodies were pre-incubated with or without each specific blocking peptide prior to Western blotting of samples from SDS fractions. **c-d**, The αSyn and p-αSyn levels in the SDS fractions were confirmed by two sets of antibodies. Both p-αSyn antibodies show similar bands between 100-150KD (red box).

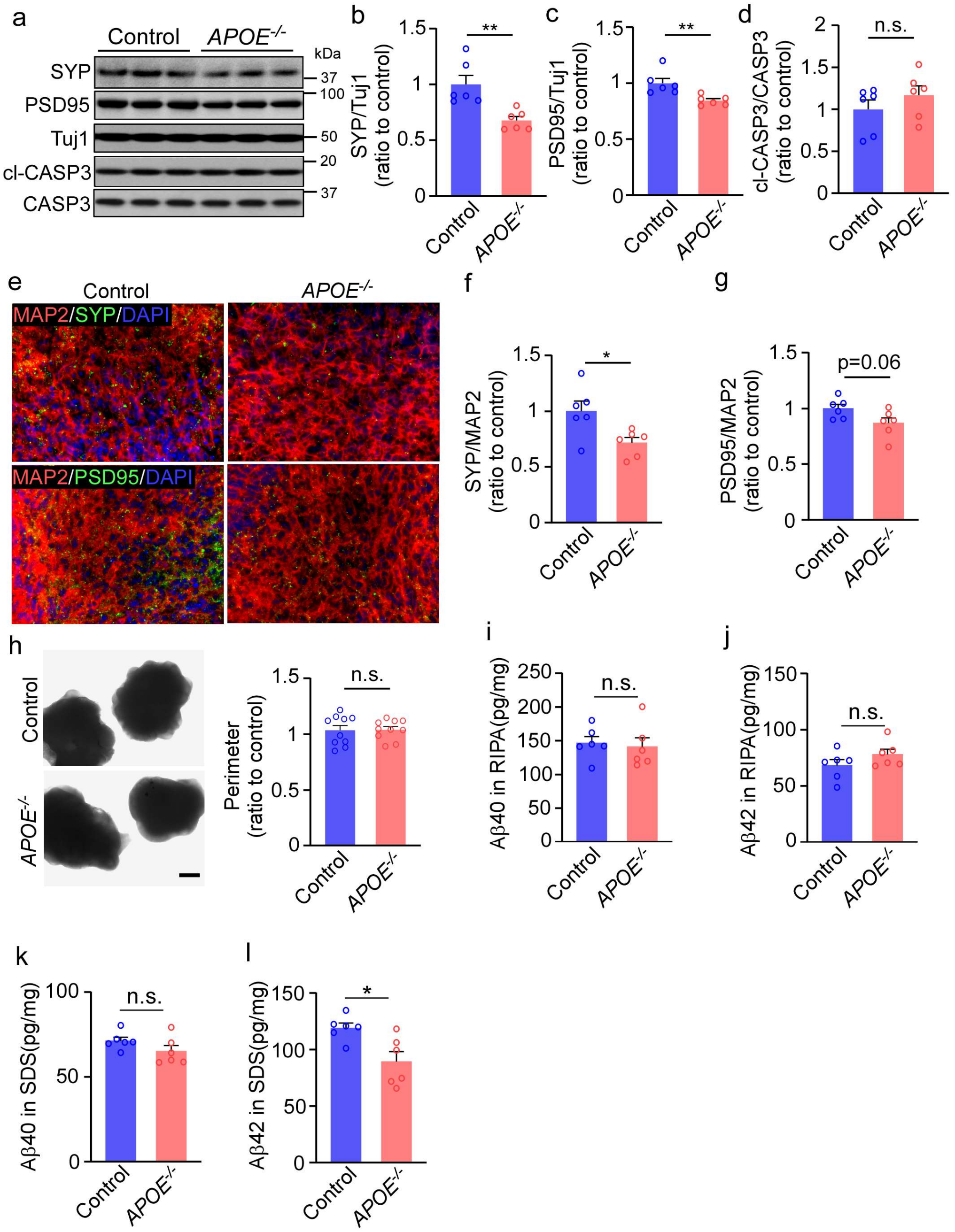

Supplementary Figure 2. Exacerbated synaptic loss and decreased insoluble Aβ42 in apoE-deficient cerebral organoids. Parent control and isogenic *APOE*$^{-/-}$ iPSCs were differentiated into cerebral organoids. **a-d**, Amounts of pre-synaptic marker synaptophysin (SYP, **b**), post-synaptic marker PSD95 (**c**), apoptotic marker cleaved-CASP3 (cl-CASP3, **d**) in the RIPA soluble fraction were quantified by Western blotting. Lysates of 3 cerebral organoids were analyzed as one sample. SYP and PSD95 levels were normalized to Tuj1 levels. Cleaved-CASP3 levels were normalized to total CASP3 levels. All data are expressed as mean ± SEM (n=6 samples/each). Experiments were repeated in three independent differentiation batches. **e-g**, The immunoreactivities of SYP (**f**) and PSD95 (**g**) in MAP2-positive neurons were quantified in the cerebral organoids by immunostaining. All data are expressed as mean ± SEM (n=6 organoids/each). **h**, Cerebral organoid perimeters were quantified at Day 90. All data are expressed as mean ± SEM (n=10 organoids/each). Scale bar: 1 mm. **i-l**, Aβ40 and Aβ42 levels in RIPA (**i**, **j**) and SDS (**k**, **l**) fractions of cerebral organoids were measured by ELISA. 3 cerebral organoids were pooled and analyzed as one sample. All data are expressed as mean ± SEM (n=6 samples/each). MannWhitney U tests were performed to determine statistical significance. **p<0.01, *p<0.05, n.s., not significant.

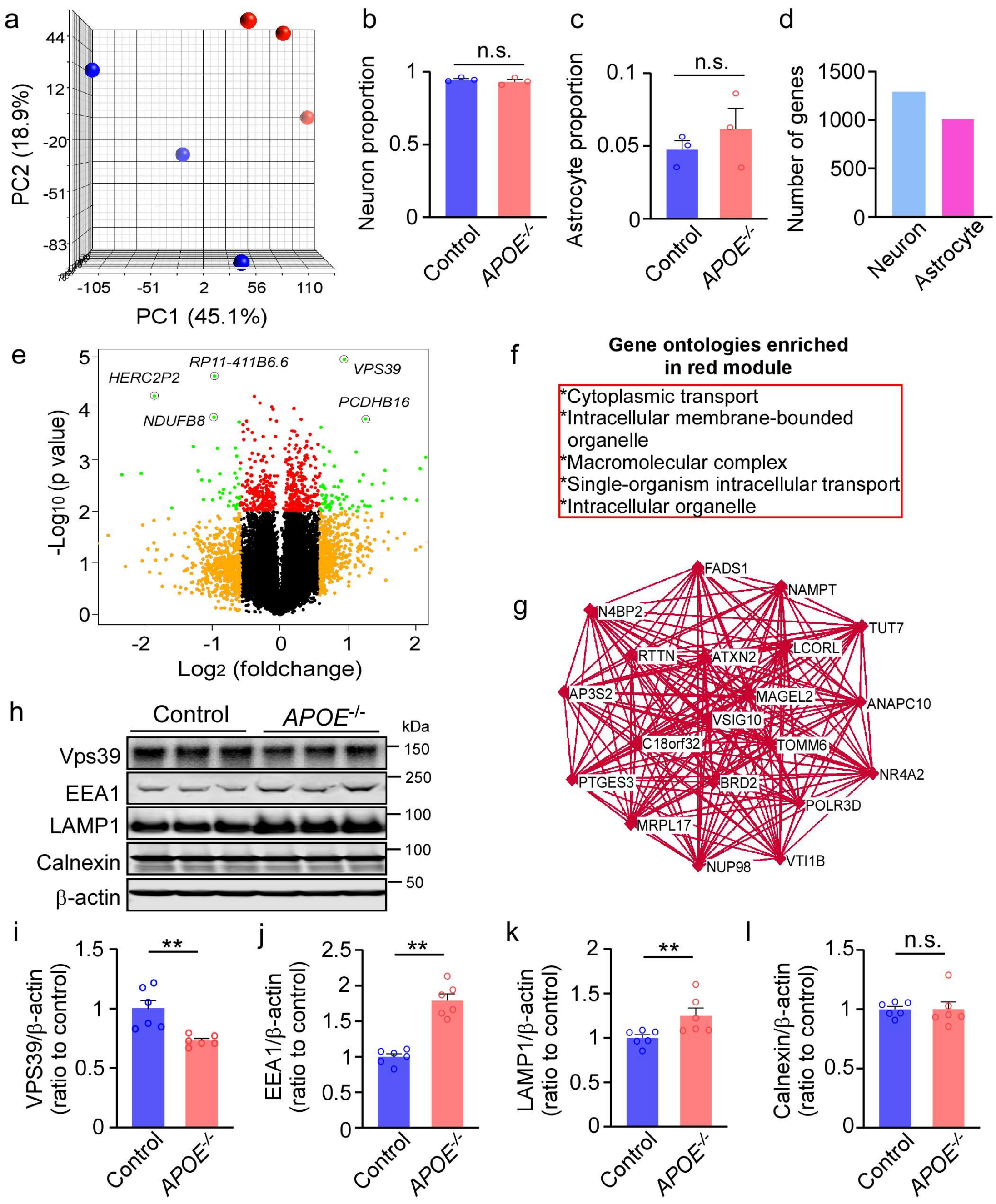

Supplementary Figure 3. Transcriptional profiling and cell composition of cerebral organoids. RNA-seq was performed on parental control and isogenic *APOE*$^{-/-}$ iPSC-derived cerebral organoids at Day 90 (n=3 samples/each). **a**, Principal component analysis (PCA) of the *APOE*$^{-/-}$ (blue) and control (red) cerebral organoids samples. **b-d**, RNA-seq data was analyzed with CIBERSORT program to evaluate the cellular composition in all samples. The proportions of neuron (**b**) and astrocyte (**c**) were compared between groups (mean ± SEM, n=3 samples/each). Numbers of DEGs assigned to neuron and astrocyte were 1297 and 1010, respectively (**d**). **e**, Volcano plots illustrating DEGs identified between control and *APOE*$^{-/-}$ iPSC-derived cerebral organoids. The green dots denote down-regulated or up-regulated DEGs (Control *vs. APOE-/-*; unadjusted P value < 0.01; ｜fold change｜>1.5). The orange dots denote the genes with P value < 0.01, but |fold change| values are less than 1.5. The black dots denote the genes with P values ⩾ 0.01, and |fold change| values are less than 1.5. Top 5 most significantly changed genes were labelled in the volcano plots. **f-g**, Top gene ontologies enriched by the red module genes revealed by WGCNA (**f**) and the interaction of top 20 genes with the highest connectivity among each other in the red module (**g**) are shown. **h-l**, Amounts of Vps39 (**i**), an endosome marker EEA1 (**j**), a lysosome marker LAMP1 (**k**) and an ER marker culnexin (**l**) in the RIPA fraction of the cerebral organoids were quantified by Western blotting. The levels of GFAP, Vps39, EEA1, LAMP1 and calnexin were normalized to β-actin. 3 cerebral organoids were pooled and analyzed as one sample. All data are expressed as mean ± SEM (n=6 samples/each). Experiments were repeated in three independent differentiation batches. Mann Whitney U tests were performed to determined statistical significance, **p<0.01.

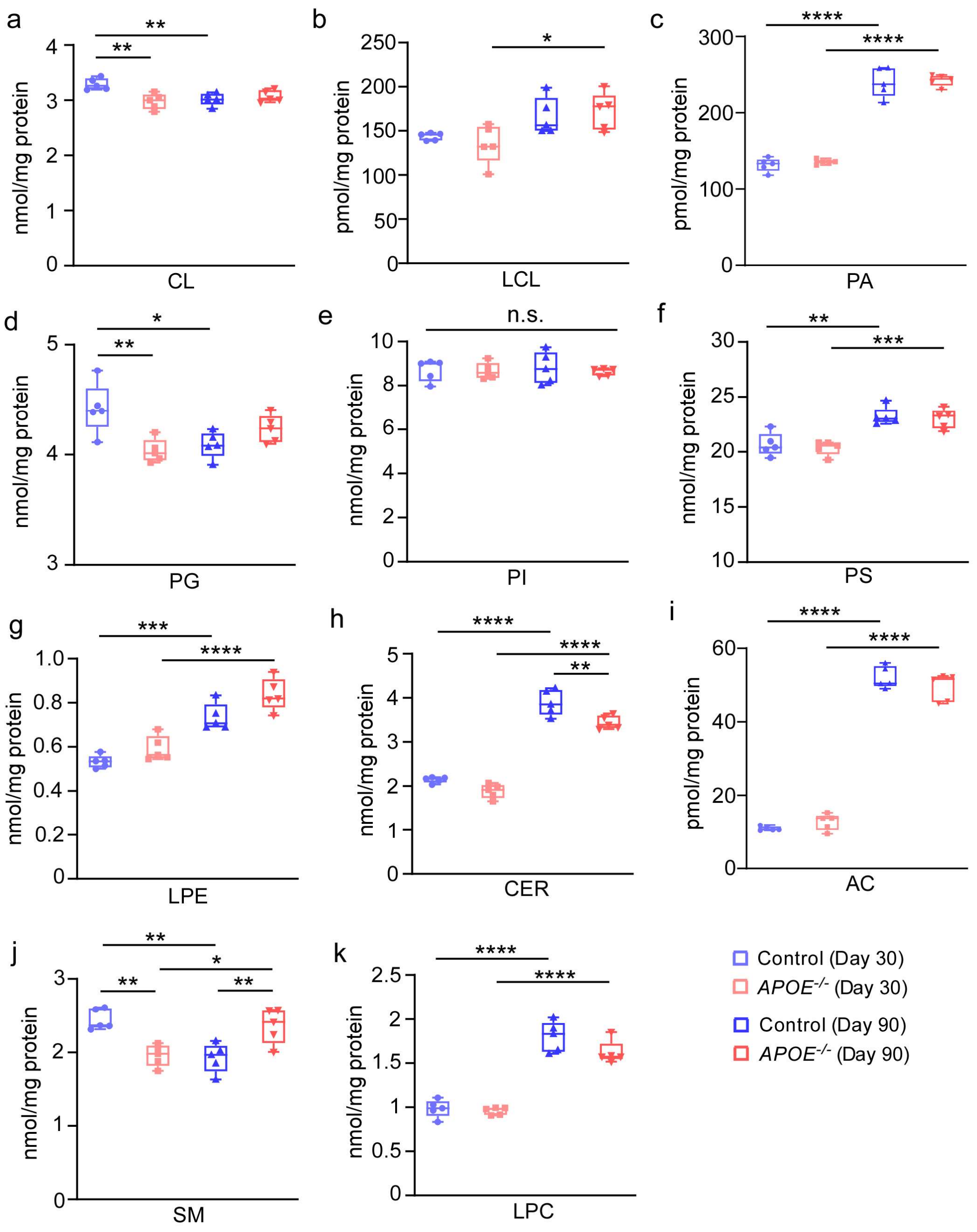

Supplementary Figure 4. Lipidomics analyses of cerebral organoids. Lipidomics was performed with lysates from parental control and isogenic *APOE*$^{-/-}$ iPSC-derived cerebral organoids at Day 30 and Day 90. Concentration of cardiolipin (CL, **a**), lyso-cardiolipin (LCL, **b**), phosphatidic acid (PA, **c**), phosphatidylglycerol (PG, **d**), phosphatidylinositol (PI, **e**), phosphatidylserine (PS, **f**), lyso-phosphatidylethanolamine (LPE, **g**), ceramide (CER, **h**), acylcarnitine (AC, **i**), sphingomyelin (SM, **j**) and lyso-phosphatidylcholine (LPC, **k**) in the lysates were plotted. All lipid concentrations were normalized to the protein levels. Lysates from 3 cerebral organoids were analyzed as one sample. All data are expressed as mean ± SEM (n=5 samples/each). Two-way ANOVA were performed to determine statistical significance. *$p<0.05$, **$p<0.01$, *** $p<0.001$, **** $p<0.0001$.

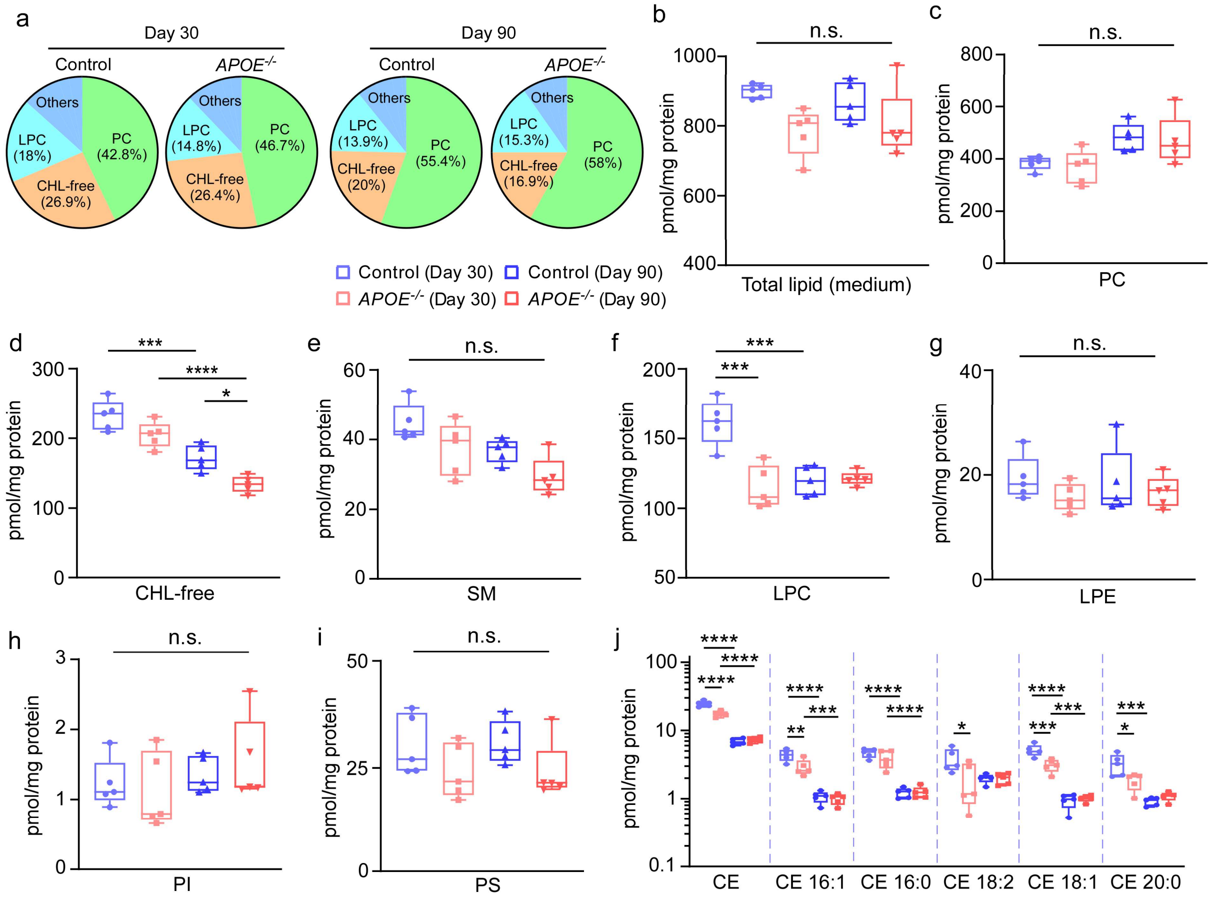

Supplementary Figure 5. Effects of apoE on lipid secretome of iPSC-derived cerebral organoids. Conditioned media (48 hours after medium change) from parental control and isogenic *APOE*$^{-/-}$ iPSC-derived cerebral organoids were collected at Day 30 and Day 90 and subjected to lipidomics and cholesterol assay. **a**, Overall composition of lipid species in the culture medium of iPSC-derived cerebral organoids. **b-j**, Concentrations of total lipids (**b**), phosphatidylcholine (PC, **c**), free cholesterol (CHL-free, **d**), sphingomyelin (SM, **e**), lyso-phosphatidylcholine (LPC, **f**), lyso-phosphatidylethanolamine (LPE, **g**), phosphatidylinositol (PI, **h**), phosphatidylserine (PS, **i**) and cholesterol ester (CE) species (**j**) in the medium were plotted. All lipid concentrations were normalized to the protein levels of cell lysates. Conditioned medium from 3 cerebral organoids were analyzed as one sample. All data are expressed as mean ± SEM (n=5 samples/each). Two-way ANOVA were performed to determine statistical significance. *p<0.05, *** p<0.001, **** p<0.0001, n.s., not significant.

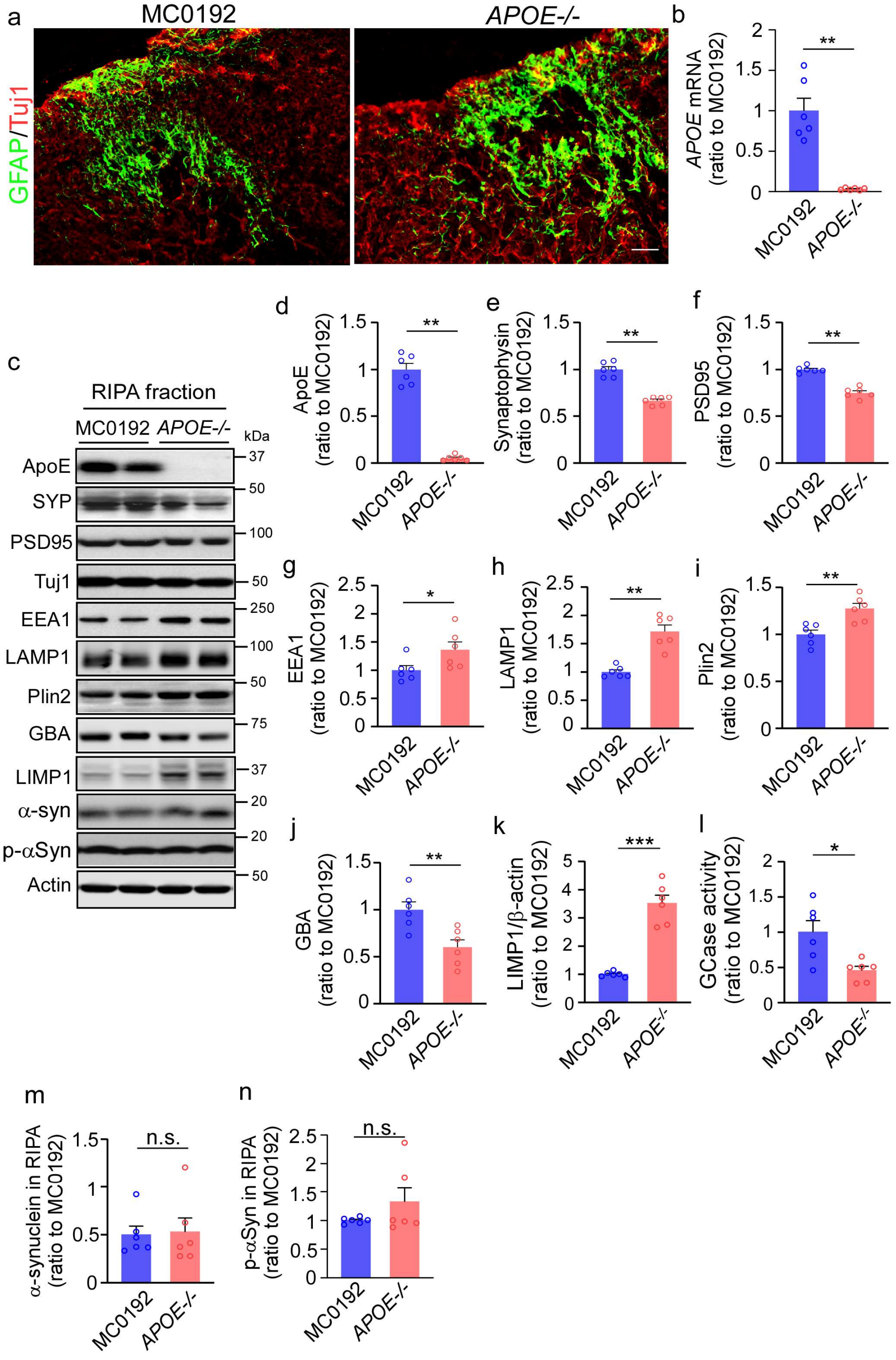

Supplementary Figure 6. Confirmation of key phenotypes associated with apoE deficiency in iPSC-derived cerebral organoids. An independent apoE-deficient (*APOE*$^{-/-}$) iPSCs were generated from a control iPSC line (MC0192). The parent control and isogenic *APOE*$^{-/-}$ iPSCs were differentiated into cerebral organoids and subjected to analyses at Day 90. **a**, Representative images of Tuj1-positive neurons and GFAP-positive astrocytes in the cerebral organoids. Scale bar: 100 µm. **b**, *APOE* depletion in isogenic *APOE*$^{-/-}$ iPSC-derived cerebral organoids was confirmed by RT-qPCR. **c-k**, **m-n**, Amounts of apoE (**d**), SYP (**e**), PSD95 (**f**), EEA1 (**g**), LAMP1 (**h**), Plin2 (**i**), GBA (**j**), LIMP1 (**k**), αSyn (**m**) and p-αSyn (**n**) in the RIPA fraction of cerebral organoids were quantified by Western blotting (**c**). ApoE, αSyn, p-αSyn, GBA, LIMP1, Plin2, EEA1 and LAMP1 levels were normalized to β-actin levels. SYP and PSD95 levels were normalized to Tuj1 levels. **l**, GCase activity in cerebral organoids was detected by GCase activity kit (Fluorometric). Data were normalized to protein concentrations. 3 cerebral organoids were pooled and analyzed as one sample. All data are expressed as mean ± SEM (n=6 samples/each). Experiments were repeated in three independent differentiation batches. MannWhitney U tests were performed to determine statistical significance. *p<0.05, **p<0.01, ***p<0.001, n.s., not significant.

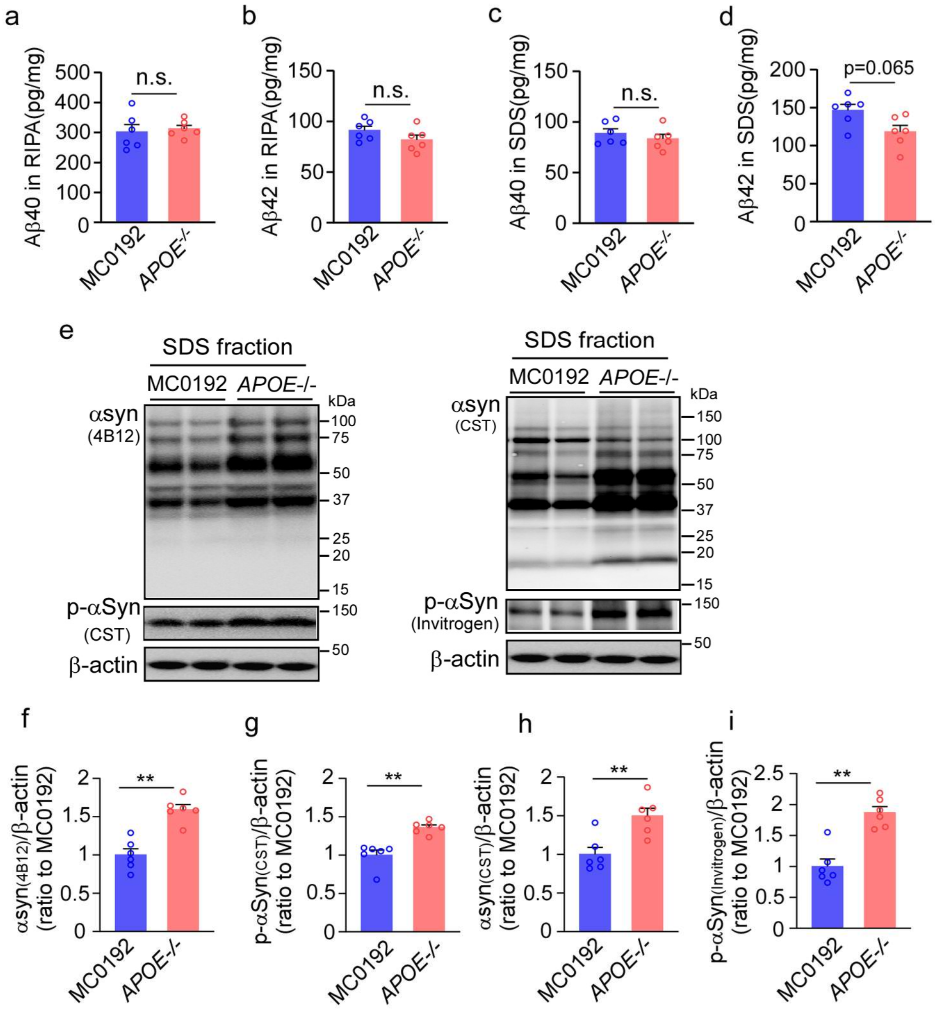

Supplementary Figure 7. Confirmation of key phenotypes associated with apoE deficiency in iPSC-derived cerebral organoids. **a-d**, Aβ40 and Aβ42 levels in RIPA (**a**, **b**) and SDS (**c**, **d**) fractions of cerebral organoids from the second set of *APOE*$^{-/-}$ isogenic iPSC lines were measured by ELISA. **e-i**, Amounts of αSyn (**f**, **h**) and p-αSyn (**g**, **i**) in the SDS fraction of cerebral organoids from the second set of *APOE*$^{-/-}$ isogenic iPSC lines were quantified by Western blotting (**e**). 3 cerebral organoids were pooled and analyzed as one sample. All data are expressed as mean ± SEM (n=6 samples/each). Experiments were repeated in three independent differentiation batches. MannWhitney U tests were performed to determine statistical significance. **p<0.01, n.s., not significant.

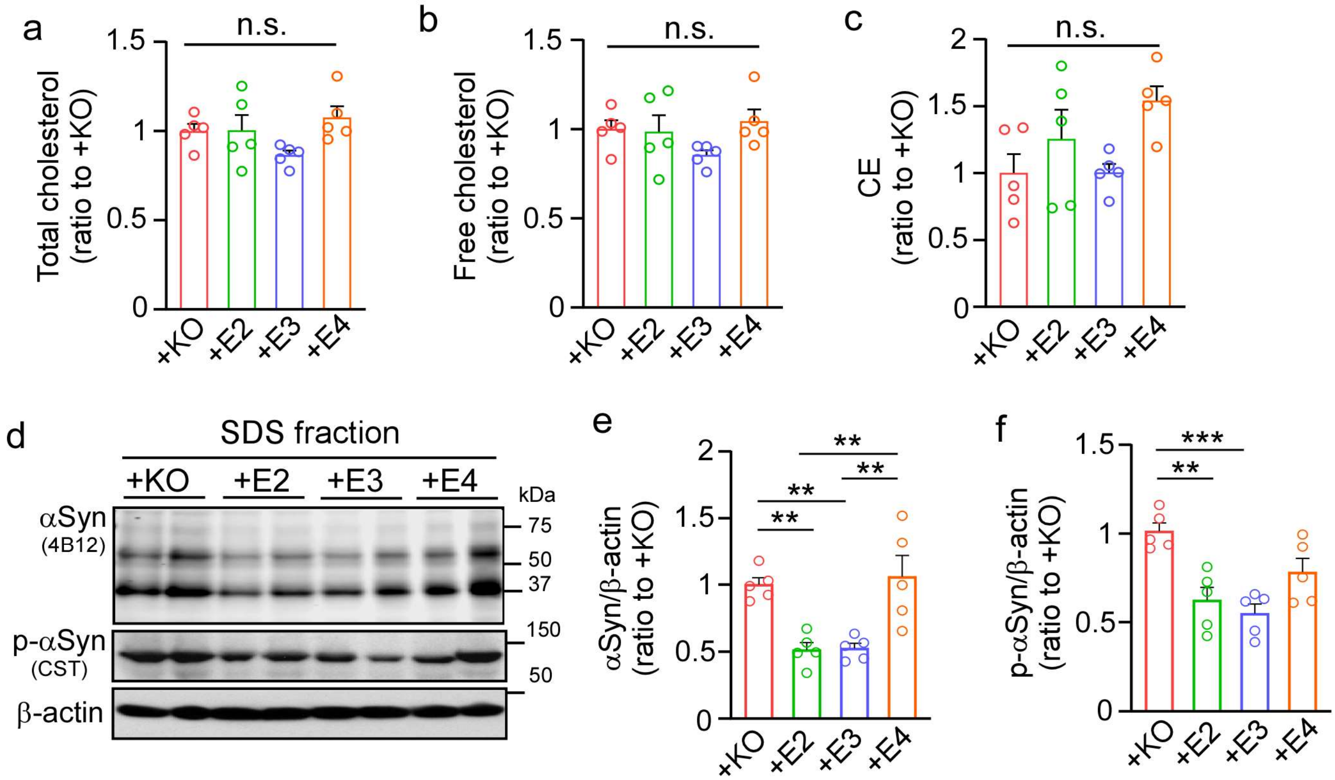

Supplementary Figure 8. ApoE2 and apoE3, but not apoE4, partially rescue pathological phenotypes in apoE-deficient cerebral organoids. The *APOE*$^{-/-}$ iPSC-derived cerebral organoids at Day 90 were treated with conditioned media of immortalized astrocytes from *APOE2*-target replacement (TR), *APOE3*-TR, or *APOE4*-TR mice for 5 days. Conditioned media from *Apoe*-KO astrocytes were used as a control. **a-c**, Amounts of total cholesterol (**a**), free cholesterol (**b**) and cholesterol ester (CE, **c**) in cerebral organoids after treatment were measured by cholesterol assay kit. Data were normalized to individual total protein concentration. **d-f**, Amounts of αSyn (**e**) and p-αSyn (**f**) in the SDS fraction of the cerebral organoids after treatments were quantified by Western blotting using another set of antibodies (**d**). All data are expressed as mean ± SEM (n=5 samples/each). Experiments were repeated in three independent differentiation batches. One-way ANOVA was performed to determine statistical significance. **p<0.01, ***p<0.001, n.s., not significant.

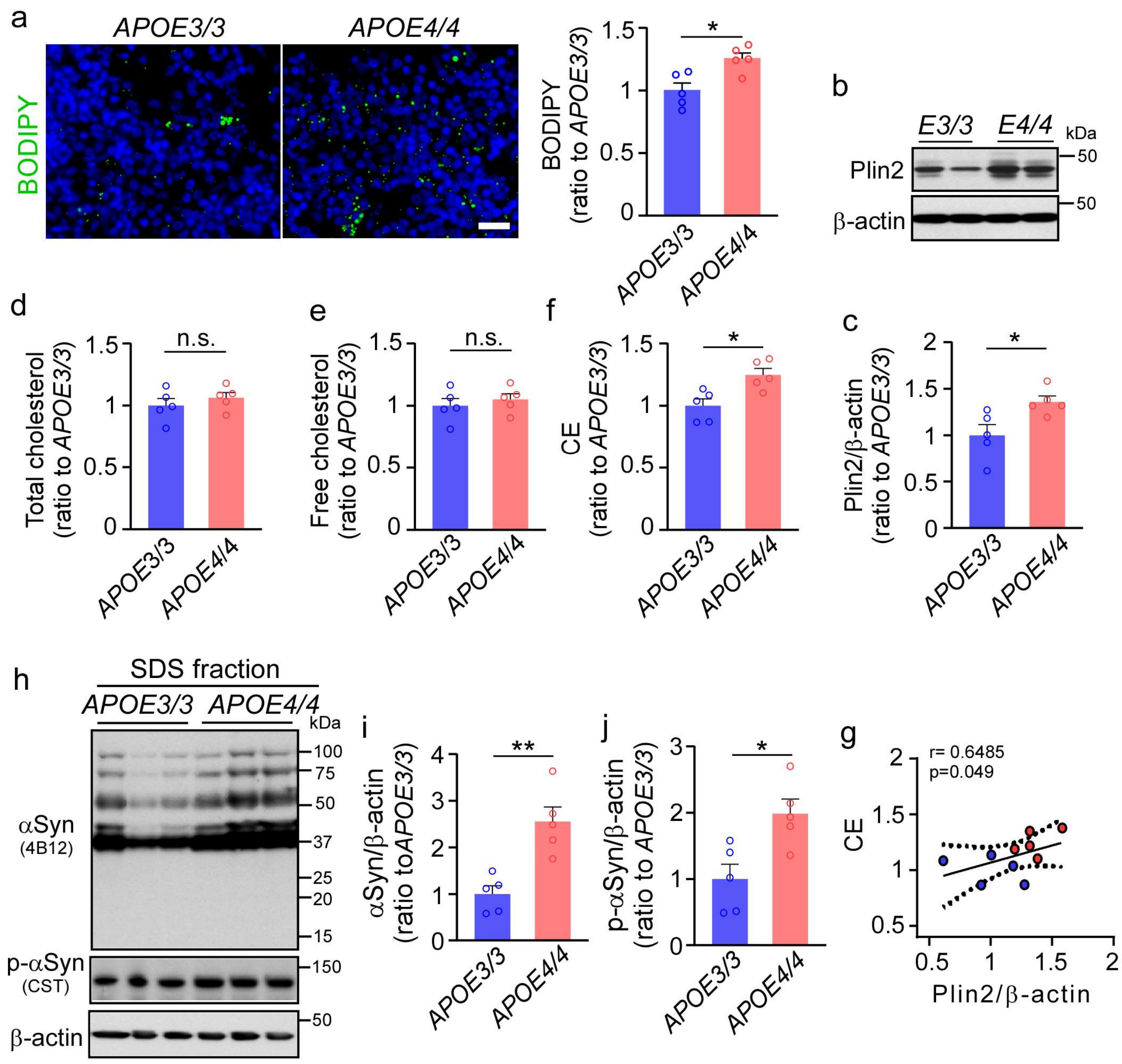

Supplementary Figure 9. Increased lipid droplet accumulation in iPSC-derived cerebral organoids with *APOE4*. Cerebral organoids were generated from iPSC lines carrying *APOE* ε3/ε3 (*APOE3/3*) or ε4/ε4 (*APOE4/4*) genotype and subjected to analyses at Day 90. **a**, Amounts of BODIPY-positive lipid droplets were quantified. Scale bar: 20 µm. **b-c**, Amounts of Plin2 in the RIPA fraction of the cerebral organoids were quantified by Western blotting. Plin2 levels were normalized to β-actin levels. **d-f**, Amounts of total cholesterol (**d**), free cholesterol (**e**) and cholesterol ester (CE, **f**) were measured by cholesterol assay kit. Data were normalized to individual total protein concentration. **g**, Spearman correlation analyses for Plin2 vs. CE in RIPA fraction are shown with the correlation coefficient (r) and the correlation p-value. Experiments were repeated in two independent differentiation batches. **h-j**, Amounts of αSyn (**i**) and p-αSyn (**j**) in the SDS fractions were quantified by Western blotting using another set of antibodies (**h**). Lysates of 3 cerebral organoids from each line were analyzed as one sample. All data are expressed as mean ± SEM (N=5 lines/each). MannWhitney U tests were performed to determine statistical significance. *p<0.05, n.s., not significant.

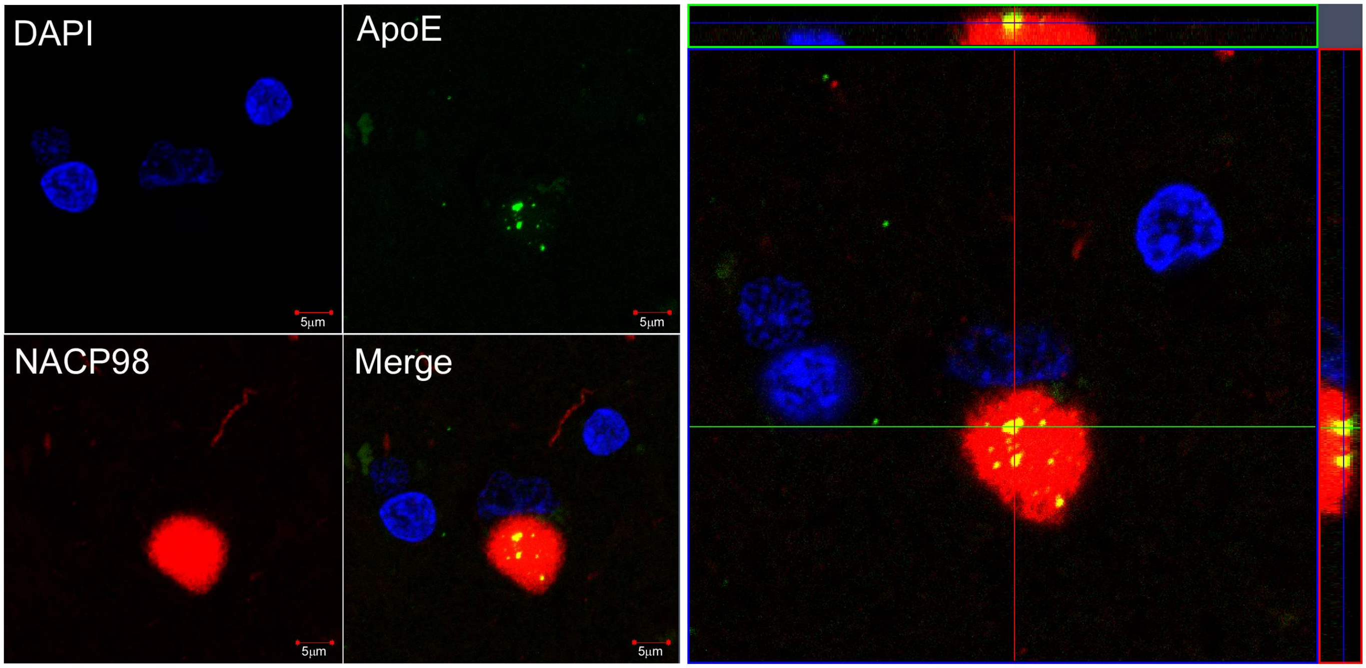

Supplementary Figure 10. Co-localization of apoE immunoreactivity with Lewy body. Human postmortem brain sections from the superior temporal cortex of LBD patients were immnunostained with an apoE antibody and α-synuclein antibody (NACP98). Scale bar: 5 μm.

Table S1. Information of iPSC lines used in this study

	ID	Sampling age	Sex	*APOE* genotype	Source	Reference
Con E3_1	mc0180	68.3	F	ε3/ε3	Fibroblasts	[4]
Con E3_2	mc0017	62.6	F	ε3/ε3	Fibroblasts	[4]
Con E3_3	mc0039	72.5	M	ε3/ε3	Fibroblasts	[4]
Con E3_4 (parental)	mc0192	83	F	ε3/ε3	Fibroblasts	[4]
Con E3_5	mc0117	71	M	ε3/ε3	Fibroblasts	[4]
Con E4_1	mc0116	83	F	ε4/ε4	Fibroblasts	[4]
Con E4_2	mc0115	87	M	ε4/ε4	Fibroblasts	[4]
Con E4_3	mc0018	67.8	F	ε4/ε4	Fibroblasts	[4]
Con E4_4	414-sc8	65	M	ε4/ε4	PBMCs	[2]
Con E4_5	384-sc4	69	F	ε4/ε4	PBMCs	[1]
XCL-1 (parental)	N/A	N/A	M	ε3/ε4	Cord blood cells CD34+	Xcell Science Inc.
XCL-KO (isogenic)	N/A	N/A	M	*APOE* knockout	Gene editing: ZFN	Xcell Science Inc.
mc0192_4C11 (isogenic)	N/A	83	F	*APOE* knockout	Gene editing: CRISPR-Cas9	[3]

Abbreviations: ZFN: Zinc Finger Nucleases; CRISPR: Clustered Regularly Interspaced Short Palindromic Repeats

Table S2. Patient characteristics for Lewy body disease cohort.

Variable	None-*APOE4* carriers (N=17)	*APOE4* carriers (N=17)
**Age at death** (mean ± SD)	72.7 ± 7.4	72.7 ± 5.8
**Sex**		
Male	9 (52.9%)	10 (58.8%)
Female	8 (47.1%)	7 (41.2%)
***APOE* genotype**		
APOE2/3	3 (17.6%)	0 (0)
APOE3/3	14 (82.4%)	0 (0)
APOE2/4	0 (0)	3 (17.6%)
APOE3/4	0 (0)	14 (82.4%)
**Thal amyloid phase** (mean ± SD)	0.8 ± 0.8	1.1 ± 0.7
**Braak NFT stage** (mean ± SD)	1.4 ± 0.9	1.4 ± 0.8
**LB count** (mean)	4.7	5

References:

1 Brookhouser N, Zhang P, Caselli R, Kim JJ, Brafman DA (2017) Generation and characterization of human induced pluripotent stem cell (hiPSC) lines from an Alzheimer's disease (ASUi001-A) and non-demented control (ASUi002-A) patient homozygous for the Apolipoprotein e4 (APOE4) risk variant. Stem Cell Res 24: 160-163 Doi 10.1016/j.scr.2017.06.003

2 Brookhouser N, Zhang P, Caselli R, Kim JJ, Brafman DA (2018) Generation and characterization of two human induced pluripotent stem cell (hiPSC) lines homozygous for the Apolipoprotein e4 (APOE4) risk variant-Alzheimer's disease (ASUi005-A) and healthy non-demented control (ASUi006-A). Stem Cell Res 32: 145-149 Doi 10.1016/j.scr.2018.09.007

3 Martens YA, Xu S, Tait R, Li G, Zhao XC, Lu W, Liu CC, Kanekiyo T, Bu G, Zhao J (2021) Generation and validation of APOE knockout human iPSC-derived cerebral organoids. STAR Protoc 2: 100571 Doi 10.1016/j.xpro.2021.100571

4 Zhao J, Fu Y, Yamazaki Y, Ren Y, Davis MD, Liu CC, Lu W, Wang X, Chen K, Cherukuri Yet al (2020) APOE4 exacerbates synapse loss and neurodegeneration in Alzheimer's disease patient iPSC-derived cerebral organoids. Nat Commun 11: 5540 Doi 10.1038/s41467-020-19264-0
